# A computational rule-based model of MAPK/ERK system regulation

**DOI:** 10.1038/s41598-026-44353-3

**Published:** 2026-03-21

**Authors:** Paweł Kocieniewski, Tomasz Lipniacki

**Affiliations:** Institute of Fundamental Technological Research, Polish Academy of Sciences, Warsaw, Poland

**Keywords:** MAPK signaling, RAF isoforms, 14-3-3 proteins, Computational model, Rule-based modeling, BNGL, Biochemistry, Biophysics, Cancer, Cell biology, Computational biology and bioinformatics, Systems biology

## Abstract

The MAPK/ERK pathway coordinates multiple cellular functions, including proliferation, apoptosis, and motility, yet it is frequently modeled as a one-purpose cascade. Pathway complexity stems from the existence of isoforms that are regulated differently and have specific interacting partners. Here, we overcome combinatorial complexity by constructing a rule-based model of the MAPK/ERK pathway that accounts for differential regulation of MEK and RAF isoforms and RAF interactions with 14-3-3 proteins. The model addresses signaling based on the enzymatic cascade as well as regulatory protein-protein interactions. We propose that at low concentrations of growth factors, RAS is activated only in a portion of the membrane. This allows the model to reconcile the observed graded and switch-like responses observed at the upper (RAS and RAF) and the lowest (ERK) tiers of the pathway, respectively. We demonstrate that functional differences between BRAF and CRAF, or ARAF, can arise from their distinct interactions with 14-3-3. The 14-3-3 dimers inhibit all RAF monomers in the closed form and preferentially stabilize BRAF-CRAF or BRAF-ARAF dimers and may not stabilize RAF dimers without BRAF. The constructed model is used to explore qualitative differences in MAPK/ERK pathway signaling across different cell lines. The exploration starts from the nominal model parameters chosen to reflect a generic cell.

**Supplementary Information:**

The online version contains supplementary material available at 10.1038/s41598-026-44353-3.

## Introduction

The MAPK/ERK pathway (also termed the Ras-Raf-MEK-ERK pathway) is a principal channel that transmits signals from growth factors, which typically promote proliferation and survival. This pathway has been intensely studied for the last 40 years due to its fundamental role in cell physiology and involvement in oncogenesis, and as such, its structure, properties, and regulation have been extensively reviewed^1–3^. In brief, growth factors such as EGF or FGF activate growth factor receptors, which dimerize and, in turn, recruit the SOS protein to the cellular membrane. SOS is a guanine exchange factor that activates a small GTPase, RAS, by stimulating the exchange of RAS-bound GDP for GTP. RAS-GTP subsequently dimerizes and recruits cytosolic RAF kinases (first-tier - MAP3K), which undergo activation through a series of phosphorylation events and conformational changes that ultimately lead to RAF dimerization and full activation. Active RAF kinases subsequently phosphorylate MEK kinases (second tier - MAP2K), which, in turn, phosphorylate ERK kinases (third tier - MAPK). The ERK kinases effectuate the physiological response by regulating hundreds of cytoplasmic and nuclear targets through phosphorylation. The ultimate nature of that response is determined by the spatiotemporal pattern of ERK activation as well as the specific set of substrates present in the cell, which depends both on the cell’s type and state^4,5^.

While the pathway is generally perceived as linear, with a simple activation flow from one tier to the next, its structure is significantly more complicated. The pathway contains numerous feedback loops. In particular, ERK mediates negative feedback to MEK, RAF, and SOS, enabling transient or oscillatory responses to sustained signals^6–8^. This feedback can reset the entire pathway and elicit pulsatile responses to both transient and tonic signals. This property allows the MAPK/ERK pathway to transmit information with high bit rates^9,10^.

In this work, we consider two MEK kinase isoforms (MEK1 and MEK2)^11^ and three RAF isoforms: BRAF, CRAF, and ARAF^12^. The three RAF isoforms share significant sequence homology and an overall layout: (1) the N-terminal autoinhibitory domain, which contains the RAS binding domain (RBD) and cysteine-rich domain (CRD) necessary for RAS and membrane recruitment, and (2) the C-terminal kinase domain; the regulatory NtA domain is located in the linker region joining the C- and N-terminal domains. Both the C- and N-terminal domains contain 14-3-3 binding sites; 14-3-3 proteins are essential regulators of RAF activity, which exist as obligatory homo- and heterodimers of seven known isoforms^13^. Each 14-3-3 protein has a highly conserved phosphorylation-binding pocket, allowing its binding to ^specific phosphorylated serine residues of RAF isoforms14^. Without growth factor signaling, all RAF isoforms exist mainly in a closed, inactive conformation where the kinase domain and CRD domain bind each other, and this autoinhibited complex is further crosslinked by 14-3-3 dimers bound to its C- and N-terminal phosphoresidues^15–17^. 14-3-3 binding at the N-terminal site interferes with RAF activation by occluding the CRD domain, which is necessary for stable binding to RAS-GTP dimers and the cell membrane. A recent Cryo-EM study showed that BRAF can bind RAS while still being in the closed conformation, implying that 14-3-3 is released from the N-terminal site upon binding Park et al.^16^ The RAF dimers may be stabilized by 14-3-3 dimers crosslinking the C-terminal phosphoresidues of the protomers. Consequently, 14-3-3 can stabilize both inactive and active RAF conformations.

The RAF isoforms differ in significant ways. BRAF is most akin to the ancestral RAF kinase; its NtA domain residues, including Ser446, are constitutively phosphorylated, which increases the basal activity of BRAF and primes it for further activation. In contrast, CRAF and ARAF require recruitment by RAS-GTP for NtA phosphorylation (at Ser338 and Ser266, respectively). Furthermore, unlike BRAF, CRAF can bind and modulate the activity of multiple other proteins through direct protein-protein interactions rather than enzymatic activity. In particular, in its closed conformation, CRAF and ARAF (when phosphorylated at Ser259 and Ser214, respectively) bind and inhibit the MST2 kinase, preventing it from participating in the pro-apoptotic Hippo pathway^14^. Engagement of ARAF and CRAF in RAS signaling releases MST2. Thus, growth factors leading to CRAF and ARAF dephosphorylation at Ser259 and Ser214 can trigger both proliferation and apoptosis, and a shift in the balance toward proliferation is associated with increased AKT activity, which inhibits MST2^18,19^. In the “open” conformation, after RAF dimer dissociation, CRAF phosphorylated at Ser338, can form complexes with ROKα, which promotes cytoskeletal rearrangements and motility^20^, and with Bcl-2, DAPK1, and ASK1, inhibiting apoptosis^21–24^.

Regarding the regulation by 14-3-3, BRAF recruits 14-3-3 primarily through its high-affinity C-terminal site (Ser729), while the lower affinity Ser365 residue on the N-terminus is considered secondary^25,26^. In turn, the primary 14-3-3 recruitment sites of CRAF and ARAF are located at the N-terminus (at Ser259 and Ser214, respectively)^14^. Consequently, the recruitment of ARAF and CRAF by RAS-GTP likely implies almost complete dissociation of 14-3-3 as the binding to the lower-affinity C-terminal sites (respectively, at Ser621 and Ser582) is unstable. Therefore, neither CRAF nor ARAF is likely to donate 14-3-3 to stabilize a RAF dimer, in contrast to BRAF, which can likely retain stable 14-3-3 binding through its high-affinity C-terminal site while fully recruited to RAS-GTP via its RBD and CRD domains.

Despite the well-defined architecture, the pathway exhibits significant variability in behavior and function across different cell lines. From a mechanistic perspective, this can be attributed to order-of-magnitude differences in the levels of expression of cascade components across cell lines.

Due to its importance, the MAPK pathway has been the subject of extensive mathematical and computational modeling to understand the roles of its structural and regulatory features in performing physiological functions. The first model was published in 1996 by Huang and Ferrel^27^. The authors demonstrated that the three-tier cascade architecture, combined with dual phosphorylation-based activation of MEK and ERK, produces ultrasensitive behavior, a finding later experimentally confirmed in Xenopus oocytes. Over the years, models steadily grew in complexity, incorporating additional features such as feedback loops, scaffold interactions, receptor activation, multisite phosphorylation, and pathway crosstalk. Feedback regulation is particularly important in shaping the ERK response time profile. The pathway contains numerous negative feedback loops, mostly based on ERK phosphorylation of upstream pathway components or the induction of negative regulators, such as phosphatases. Modeling studies have demonstrated their roles in limiting the response (transient vs. sustained)^28^ and potentially inducing oscillations^29^. Sturm et al. have also demonstrated that negative feedback can endow the cascade with properties of a negative feedback amplifier, which increases its robustness to internal and external perturbations^30^.

The ERK pathway also contains positive feedback loops, whose functions were predicted to potentiate and sustain ERK activation by weak signals^31^, which explain observed switch-like activation of ERK^32^, and potentially introduce bistability. Xiong et al. have indeed experimentally verified that a positive feedback loop (ERK-Cdc2) underlies Xenopus oocyte maturation and concomitant all-or-none activation of ERK^33^; they have also demonstrated the system to be bistable due to the combination of this positive feedback loop with the non-linear activation of ERK, providing a basis for irreversible cell fate decisions and memory. Another positive feedback loop is based on the upregulation of SOS GEF activity by allosteric binding of RAS-GTP^34^. This feedback was theoretically demonstrated to drive, in the case of slow diffusion, membrane clustering and activation of traveling waves^35^; it was experimentally shown to drive digital, hysteretic RAS activation in lymphoid cells^36^.

Computational studies also uncovered non-obvious sources of positive feedback that do not require explicit upregulation but emerge from substrate-based enzyme saturation or sequestration. In particular, Markevitch et al. demonstrated an effective positive feedback based on the dual (de)phosphorylation cycle of the enzymes in the cascade, which can yield bistability^37^. They then demonstrated how this mechanism could underlie long-range propagation of ERK activity in the cell^38^. Legewie et al. later proposed another model with positive feedback stemming from enzyme-substrate interactions, where fully phosphorylated ERK dissociates from MEK and increases its fraction available for activation by RAF^39^.

Combinations of negative and positive feedback potentially produce even more complex behavior. Kochańczyk et al. 2017 have demonstrated how a fast SOS-RAS positive feedback loop embedded in a slow negative feedback loop (ERK to SOS) can produce relaxation oscillations and enable frequency rather than amplitude-based encoding^40^. Marrone et al. have shown more generally how positive and negative feedback jointly ensure either (1) relaxation-type oscillations or (2) smoother oscillations in a narrow range of frequencies^41^.

Few models have addressed the role of isoforms in the cascade. Harrington et al. demonstrated that different trafficking constants between active ERK1 and ERK2 can affect their intracellular distribution (cytoplasm vs. nucleus) and, therefore, the cell response^42^. In another early model, Robubi et al. incorporated the then-known differences between BRAF and CRAF in their (in)activation rates and enzymatic activities; the model suggested that both isoforms equally affect the response amplitude, but BRAF is more responsible for the response duration^43^. Subsequently, Kocieniewski and Lipniacki accounted for the differences in negative feedback regulation of MEK1 and MEK2. They demonstrated that the amplitude of the ERK response can be controlled by the total amount of MEK1 and MEK2 while their ratio controls the response duration^44^.

In this study, following our earlier models (Kochańczyk et al. 2017 and Varga et al. 2018), we construct a rule-based MAPK system model that captures the combinatorial complexity of RAF isoform signaling in the MAPK/ERK system^26,40^. We exploit the model to investigate (1) switch-like and gradual activation of respectively the lowest (ERK) and the upper (RAS and RAF) tiers of the signaling cascade, (2) regulation of CRAF competence to interact with ROKα, and MST2, and (3) differential regulation of BRAF, CRAF, and ARAF isoforms by 14-3-3 proteins.

## Results

### Rule-based model of MAPK pathway

The proposed model expands the MAPK pathway model constructed by Kochańczyk et al. 2017 and later developed by Varga et al. 2017 to elucidate the dynamics of CRAF-ROKα interactions^26,40^. Following the original model, the new model accounts for one positive feedback from RAS to SOS that is responsible for bistability (observed in the absence of negative feedbacks), and three negative feedbacks mediated by ERK and involving MEK1 (and indirectly MEK2), RAFs, and SOS. The last feedback loop from ERK to SOS is of key importance as it allows for relaxation oscillations, while the feedback loops to MEK and RAFs mainly modulate the shape of these oscillations.

The model is focused on the specificity of MEK and (mainly) RAF isoforms regulation and their complex interactions with 14-3-3 proteins. To account for these interactions, we employed rule-based modeling that allows the definition of systems with a large number of species (for example, phosphostates of proteins and their complexes) that are regulated by a large number of biochemical interactions using a smaller number of rules^45,46^. In the study, we do not aim to fit parameters to reproduce the behavior of cells of any particular type but instead use the proposed model as a platform to investigate qualitative changes in cellular responses that may result from changes in particular parameters. The model parameters were chosen to reflect the generic cell; these nominal values are important because, for technical reasons, they influence the explored parameter combinations.

**Fig. 1:**
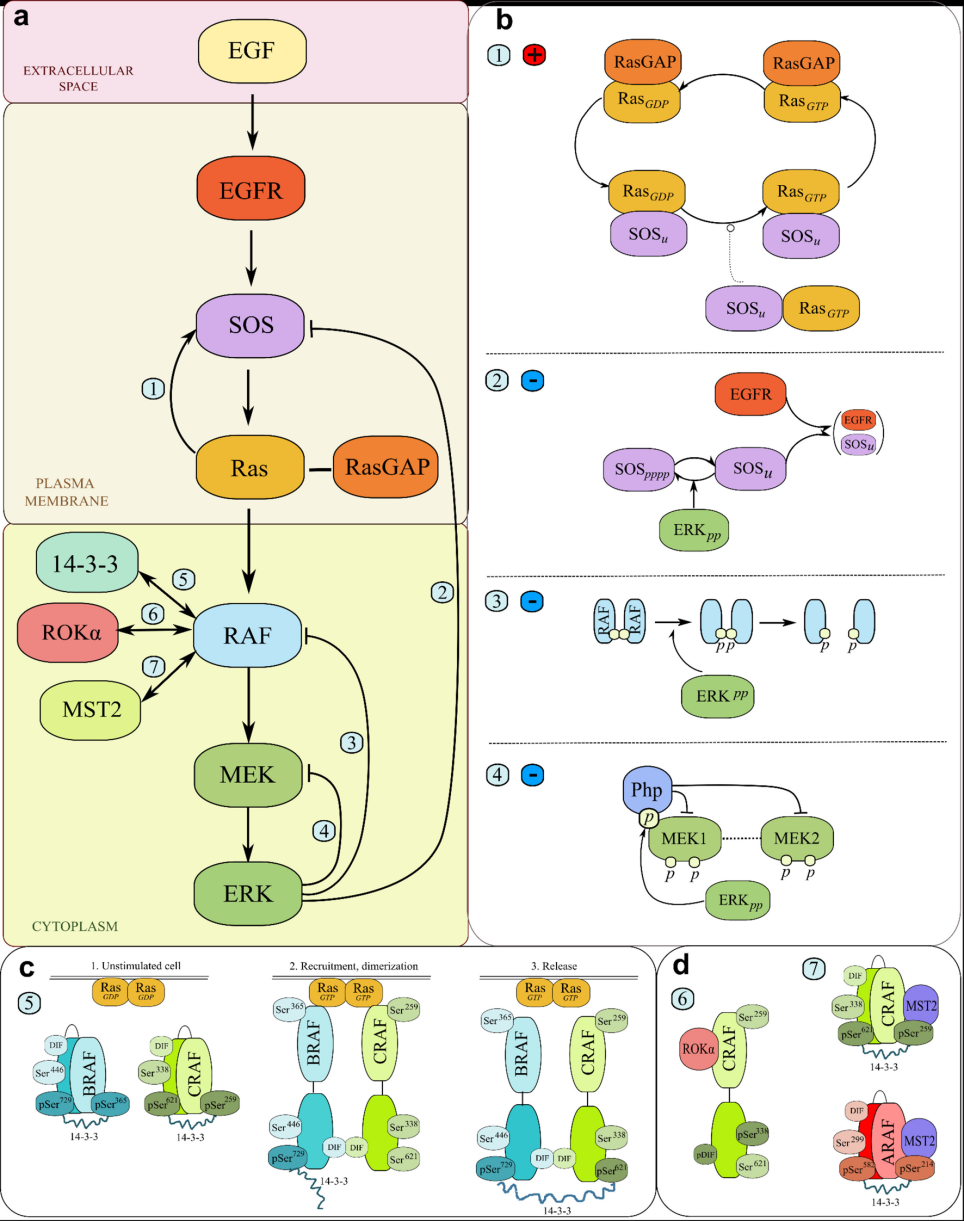
Scheme of the model. (**a**) The diagram of the RAF/ERK pathway. The model accounts for MAPK/ERK pathway signaling from the level of extracellular EGF stimulation through the distinct membrane and cytoplasmic compartments. It comprises all the tiers of the cascade down to ERK and includes three RAF isoforms (BRAF/CRAF/ARAF) as well as two isoforms of MEK (MEK1/MEK2). (**b**) The feedback loops architecture. The model accounts for one positive feedback loop (1) SOS-RAS and three negative feedback loops from ERK-PP to (2) SOS, (3) RAF, and (4) MEK. (**c**) The RAF kinases protein-protein interactions. The model accounts for interactions of the RAF isoforms with 14-3-3 proteins. In particular, BRAF initially binds 14-3-3 via its C-terminus, while CRAF and ARAF bind via their N-terminus. In quiescent cells, all RAF kinases exist in the “closed” conformation, crosslinked by 14-3-3. In stimulated cells, RAF kinases in the “open” conformation without 14-3-3 bound to their N-termini are recruited by RAS-GTP platforms and form homo- and heterodimers. Since BRAF can retain 14-3-3 at its C-terminus, 14-3-3 can crosslink BRAF with CRAF, ARAF, or other BRAF, stabilizing B-B, BC, and BA dimers. In contrast, in the model CC, AA, and CA homo- and heterodimers cannot be stabilized via 14-3-3 crosslinking. (**d**) The CRAF and ARAF protein-protein interactions. When CRAF is in its open conformation and phosphorylated on the NtA domain (Ser338), it can bind and inhibit ROKα); feedback phosphorylation at the dimerization interface (DIF) by ERK-PP disrupts dimers and enables this interaction (6). When phosphorylated at their N-terminal 14-3-3 binding sites (Ser259 and Ser214), both CRAF and ARAF can bind and sequester MST2 kinase (7)

The key (model) interactions between MAPK pathway components are visualized in Fig. 1. The details of the structural organizations of RAF, MEK, and ERK kinases, based on their crystal structures, are discussed in Nussinov et al. 2025^47^. RAF kinases activate after dimerization upon binding to membrane RAS-GTP dimers. MEK1 and MEK2 dimerize and are activated by distributive phosphorylation catalyzed by RAFs at Ser218/222 and Ser222/226, respectively. MEK1 can be phosphorylated at the Thr292 residue by ERK, which presumably creates a binding site for a phosphatase-containing complex that dephosphorylates both MEK1 and MEK2^48,49^. ERK has two isoforms, ERK1 and ERK2 (not distinguished in the model), that are activated by distributive phosphorylation by MEK1/2 at Thr202/Tyr204 and Thr185/Tyr187, respectively. The BioNetGen specification of the computational model is provided as Supplementary Code S1. The species specification and nominal parameters are summarized in Supplementary Text S1. The protein abundances are assumed in agreement with PaxDB Protein Data Base and sources within^50–58^. The details of the model structure and numerical simulations are provided in Materials and Methods.

### Bistability and relaxation oscillations

**Fig. 2:**
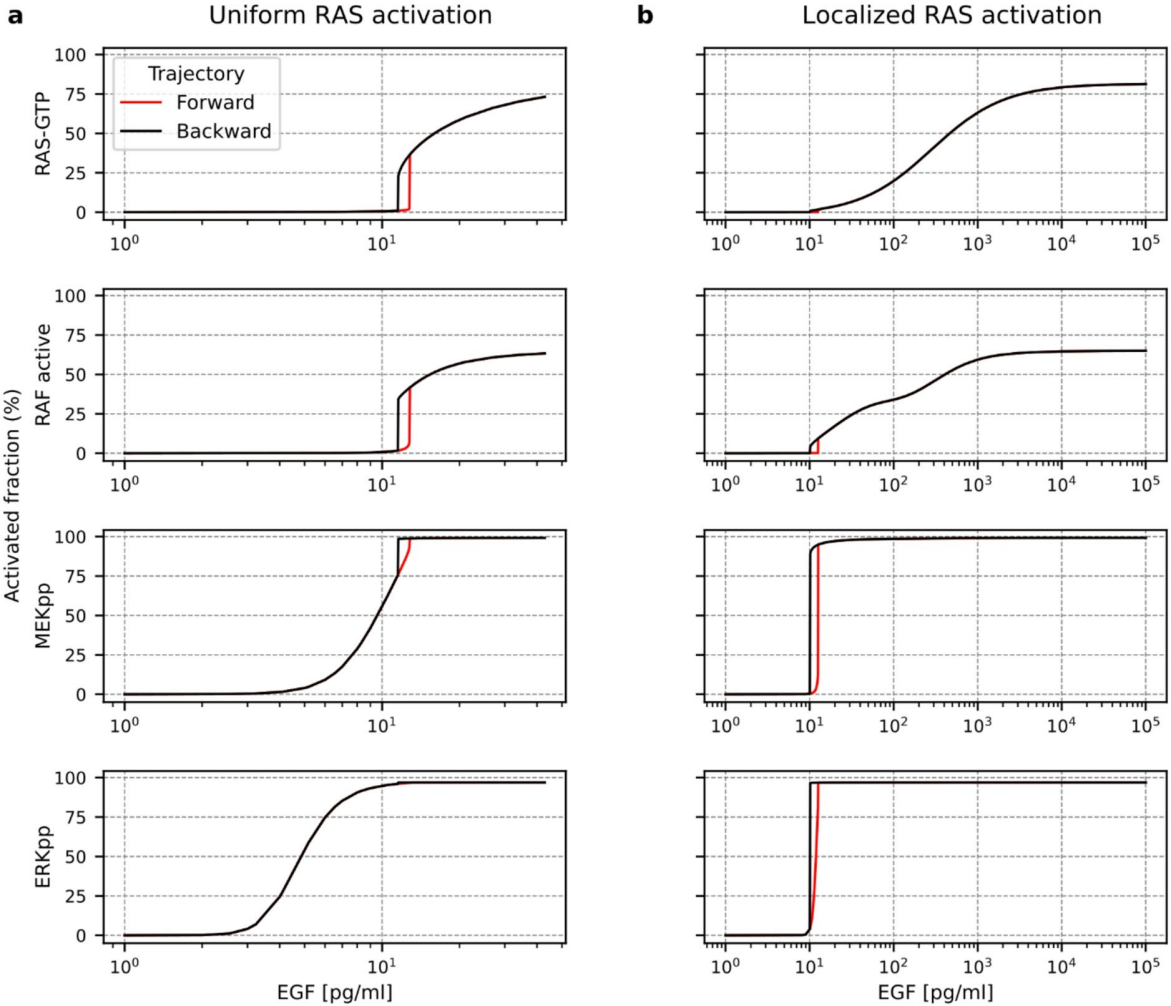
Bistable switching with all negative feedback loops removed. Forward and backward activation profiles of RAS-GTP, active RAFs, MEK-PP, and ERK-PP in response to, respectively, increasing and decreasing concentration of EGF. Active RAF stands for all dimerized RAF isoform molecules that are phosphorylated at both their NtA domains and C-terminal 14-3-3 binding sites. **(a)** For uniform RAS activation, an abrupt switch is observed for RAS-GTP and active RAF. **(b)** For RAS activation restricted to a portion of the membrane (growing with EGF concentration), an abrupt switch is observed for MEK-PP and ERK-PP. Notice the different horizontal scales in the two panels.

As in the Kochańczyk et al. 2017 model^40^, system bistability, observed in the absence of negative feedbacks, is a consequence of positive feedback coupling SOS and RAS together with nonlinearity in RAS-GTP to RAS-GDP conversion associated with low abundance of the ‘converting enzyme’ RAS-GAP. Such architecture results in switch-like responses of the upper tiers of the signaling cascade: RAS and RAF, meaning an abrupt growth of activity of these components from the low to the high levels (Fig. 2a). Experimental data indicate, however, that although ERK activation has typically a switch-like character^32^, the activity of RAS and RAF (when observed at the whole cell level) grows more gradually with signal strength^19,20^. To reconcile these observations, we propose that at low EGF concentration, switch-like activation of RAS occurs only on a fraction of the cellular membrane, *f*, which increases with EGF concentration. We chose *f* = *EGF*/(*EGF +* M_EGF_) with M_EGF_ = 300 pg/ml; the M_EGF_ value defines the EGF range for which gradual growth RAS and RAFs activity is observed, which can vary between cell lines. Such localized RAS activations are observed experimentally in response to various stimuli and enable RAS to coordinate cytoskeletal reorganization, cell-cell adhesion, and, eventually, cell motility^59,60^. As the bistable RAS switch occurs only on a portion of the membrane, at the whole cell level it produces just a small jump of RAS activity (Fig. 2b). However, because of signal amplification at the RAF, MEK and ERK levels, activation of a relatively small fraction of RAS leads to nearly full activation of MEK and ERK; when RAS switches to its upper branch, the doubly phosphorylated MEK and ERK (MEK-PP and ERK-PP) reach more than 90% of the whole protein levels. Consequently, we observe nearly gradual activation of RAS and RAF and switch-like activation of ERK (Fig. 2b). Because of high signal amplification downstream of RAS, its oncogenic mutations that lead to its constitutive activation are difficult to counteract at lower tiers of the pathway.

**Fig. 3:**
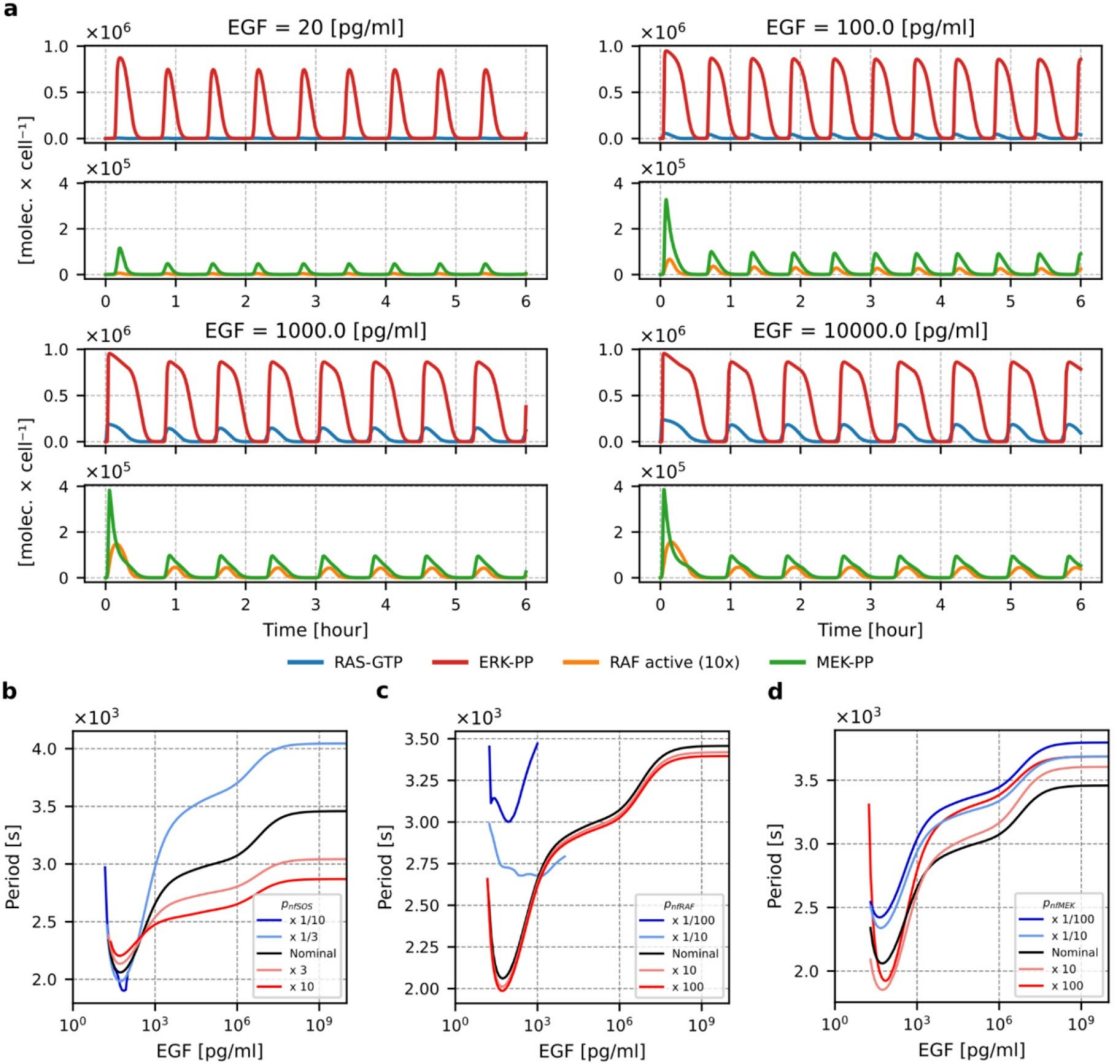
Relaxation oscillations of the MAPK system. (**a**) Relaxation oscillation profiles of RAS-GTP, active RAF, MEK-PP, and ERK-PP for four EGF concentrations as indicated. Notice the different vertical scales in the subpanels. (**b**), (**c**) and (**d**) Oscillation period as a function of EGF concentration for different strengths of the negative feedback from ERK to SOS (**b**), ERK to RAF (**c**), and ERK to MEK (**d**). The ends of the colored lines denote the critical EGF values between which limit cycle oscillations exist. The black lines correspond to the nominal values of the feedback strengths: ${p}_{nfSOS}$ = $3\times10^{-8}{[molec\times{s}]}^{-1}$, ${p}_{nfRAF}$ =$3\times{10}^{-8}{[molec\times{s}]}^{-1}$, ${p}_{nfMEK}$= $3\times{10}^{-9}{[molec\times{s}]}^{-1}$. For the ERK-to-SOS feedback 10 times lower than the nominal value, limit cycle oscillations are observed only for low EGF concentrations below 80 pg/ml. For the weak negative feedback from ERK to RAF (10 and 100 times lower than the nominal value), oscillations have a much lower amplitude, and for some range of EGF concentration, period doubling is observed. The range of limit cycle oscillations is also limited.

In both model variants, the bistability region is relatively narrow and extends for EGF concentrations between approximately 10 and 12 pg/ml. From now on, we focus on the (nominal) model in which RAS activation is localized to a portion of the cell membrane. The negative feedback from ERK to SOS encompasses the positive feedback from SOS to RAS and frustrates the bistability to produce relaxation oscillations, Fig. 3a. Not surprisingly, the level of RAS controls the existence and amplitude of oscillations, as analyzed in the Supplementary Text 1 (with Supplementary Figs. S1 and S2). Oscillations are possible in a certain region of the RAS level. For the nominal model parameters, limit cycle oscillations exist for EGF concentrations exceeding ~ 14 pg/ml. As shown in Fig. 3b for the nominal model parameters, the period is shortest for EGF concentrations around 50 pg/ml and then increases with the strength of stimulation.

The oscillation period (and range of EGF concentrations for which limit cycle oscillations are observed) depends on the strength of the negative feedback from ERK to SOS (Fig. 3b) and from ERK to RAF (Fig. 3c), while the influence of the strength of ERK to MEK feedback is much weaker (Fig. 3d). For tenfold weaker feedback to either SOS or RAF the limit cycle oscillations are observed for low EGF concentrations only. This implies that cells of a particular type (and thus feedback strength) can be tuned to respond in an oscillatory manner to EGF stimulation up to some arbitrary concentration. As temporal response patterns dictate physiological outcomes (such as proliferation or differentiation^61^, the same stimuli can lead to different outcomes depending on the cell type^5^.

The dependence of the first and subsequent peak amplitudes on EGF concentration differs for the four MAPK/ERK cascade tiers, Fig. 3a. The amplitude of ERK-PP oscillations remains nearly independent of EGF concentration, and the first peak is only somewhat higher than subsequent ones. Nearly all-or-none ERK activation is crucial for whole-system regulation, as it allows ERK to terminate both weak and strong signals. The MEK-PP time-profile remains nearly unchanged for EGF concentrations higher than 100 pg/ml, with the first peak amplitude higher than the amplitudes of subsequent peaks. With respect to this, RAS coordinates both ERK and AKT activity as well as cell motility; CRAF, depending on its phosphorylation status, may form complexes with ROKα and ASK1, regulating cell motility and inhibiting apoptosis; and both ARAF and CRAF form complexes with MST2, regulating apoptosis^18,19,26^.

### Activation of RAS, ERK proteins, RAF, and MEK isoforms

**Fig. 4:**
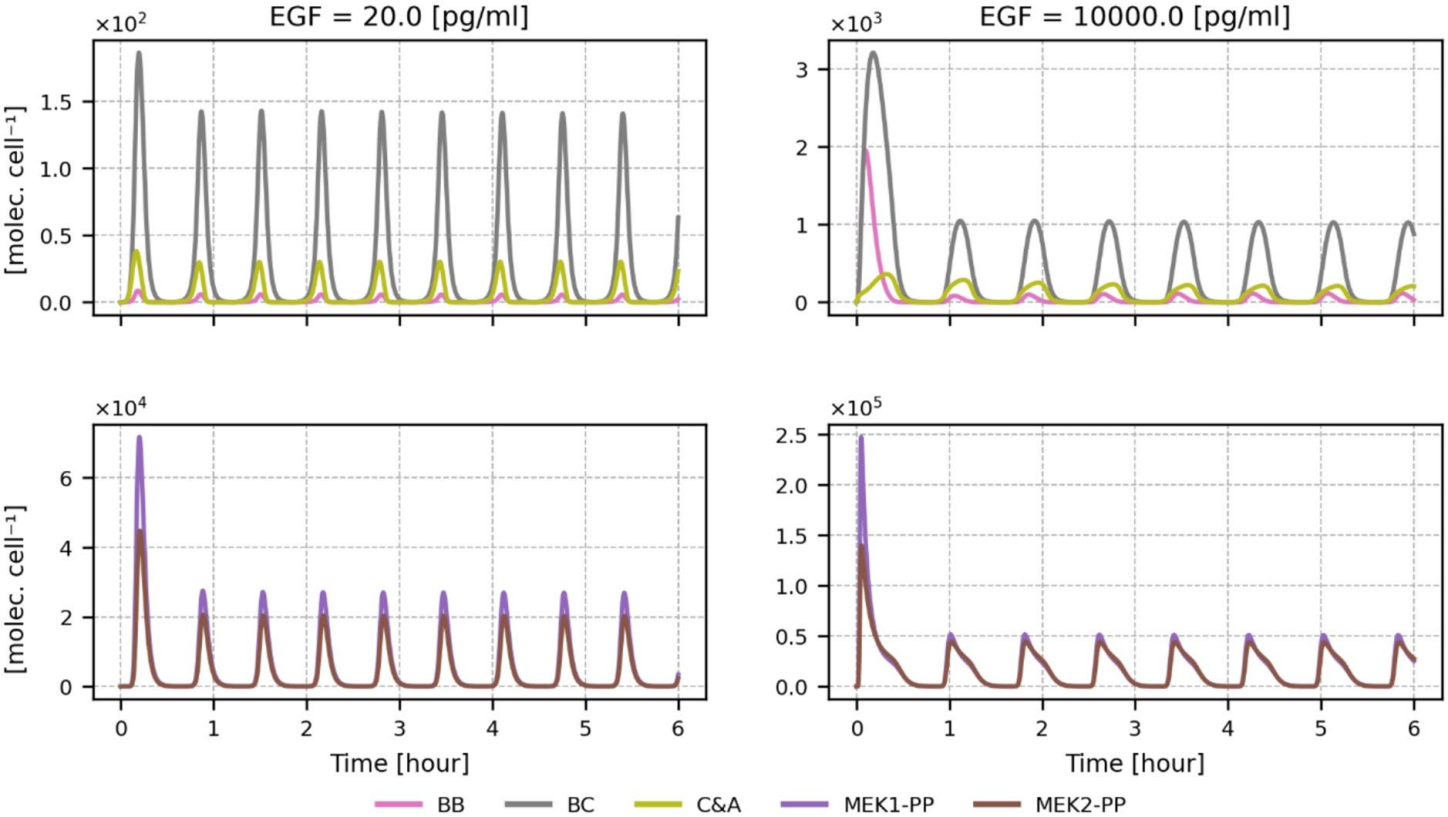
Relaxation oscillation profiles of RAF and MEK isoforms. The plot shows abundances of BB, BC, and C&A RAF dimers, and MEK1-PP & MEK2-PP molecules for low and high EGF concentrations. C&A dimers stand for all RAF dimers that do not contain BRAF. The corresponding plots for intermediate EGF concentrations of 100 pg/ml and 1000 pg/ml are provided in Supplementary Figure S5.

The existence of three RAF and two MEK isoforms implies that the signal from RAS to ERK has several paths to follow. Although only MEK1 possesses the ERK phosphorylation site (Thr292) and mediates negative feedback from ERK, the activation profiles of MEK1 and MEK2 are nearly identical (Fig. 4). This is because ERK-phosphorylated MEK1 may bind a MEK-specific phosphatase that dephosphorylates MEK1 as well as MEK2 when the latter is in a heterodimer with MEK1. See Supplementary Text (with Supplementary Figs. S3 and S4) for a detailed analysis of pathway regulation by the MEK isoforms.

In turn, RAF isoforms, which may form both homodimers and heterodimers, are regulated distinctively (Fig. 4 and Supplementary Fig. S5). For the sake of simplicity, we have assumed the same rules and constants for CRAF and ARAF regulation, but different rules for BRAF. The stoichiometry of RAF isoforms varies pronouncedly between cell lines^50^, with BRAF being the most abundant in the brain and the least abundant in most other tissues (see also Supplementary Table 1 for other protein abundances). In the model, we assume that BRAF: CRAF: ARAF remain in the proportion 2:5:5, unless otherwise specified. The difference between BRAF and the other two isoforms is in their interaction with 14-3-3 dimers. To recapitulate, in the absence of a signal, all three isoforms are mainly in their closed autoinhibited forms, being crosslinked by 14-3-3 dimers, which are bound to their (phosphorylated) N- and C-terminal phosphosites. In turn, when activated, RAF dimers may be stabilized by 14-3-3 dimers bound to the C-terminal phosphosites of the RAF protomers.

The difference between RAF isoforms follows from the fact that the primary binding site for 14-3-3 on BRAF (Ser729) is on its C-terminal, while the residue on the N-terminus (Ser365) is considered secondary. In contrast, for CRAF and ARAF, the primary 14-3-3 binding sites (respectively Ser259 and Ser214) are on the N-terminus, while secondary binding sites (Ser582 and Ser621) are on the C-terminus^14,18,26^. We have previously demonstrated that CRAF S259A substitution prevents CRAF-14-3-3 complex formation^26^. To reflect the lower affinity of binding to secondary sites, it is assumed that when 14-3-3 binds a RAF monomer, it must first bind the stronger primary site, then the secondary, and when it dissociates, it first dissociates from the secondary site. The three RAF isoforms bind RAS via their N-terminal sites. In light of these simplifying assumptions, in our model CRAF and ARAF can bind RAS only when their N-terminal sites are not bound to 14-3-3, which implies that 14-3-3 is dissociated also from the secondary sites on the C-terminus.

BRAF can bind RAS, having a 14-3-3 dimer bound to its C-terminal site, and this fact has pronounced dynamical consequences: The 14-3-3 dimer associated with BRAF can then bind the C-terminal sites of CRAF, ARAF, or other BRAF stabilizing BRAF-CRAF (BC) or BRAF-ARAF (BA) heterodimers or BRAF-BRAF (BB) homodimers (provided that the C-terminal site on the second BRAF is free). This difference implies that BC, BA, or BB dimers can be stabilized by 14-3-3. The other dynamical consequence is such that it is easier for BRAF (than CRAF or ARAF) to bind RAS because when BRAF “breathes” (i.e., switches between its closed and open conformations), it exposes the RAS binding site, while CRAF or ARAF “breathe”, exposing their C-terminal sites. Consequently, as shown in Fig. 4, BC heterodimers are much more abundant than all other dimers that do not contain BRAF (i.e., CC, AA, and CA, jointly termed C&A dimers), in spite of BRAF being less abundant than CRAF and ARAF.

BC heterodimers are also more abundant than BB homodimers. The main reason is that CRAF is assumed to be more abundant than BRAF. As said, BRAF can bind RAS more easily than CRAF (and ARAF), but on the other hand, there is a higher chance for a BC heterodimer than a BB homodimer to be stabilized by a 14-3-3 dimer, as this requires dissociation of 14-3-3 from one of the C-terminal sites before the dimer dissociates. These two opposing effects are well visible at the first peak for high EGF concentration in Fig. 4. The relative height of the peak for BB versus BC dimers is greater than predicted by stoichiometry, but the BB peak is narrower. The described rules governing RAF isoforms 14-3-3 interactions cause that at EGF concentrations higher than 100 pg/ml (Supplementary Fig. S5 and Fig. 4), the first peak amplitude is higher for BB homodimers than for all C&A dimers combined. For low EGF concentrations, as well as in subsequent activity peaks, C&A dimers are more abundant than BB homodimers, because CRAF and ARAF outcompete BRAF for binding the small pool of RAS-GTP.

**Fig. 5:**
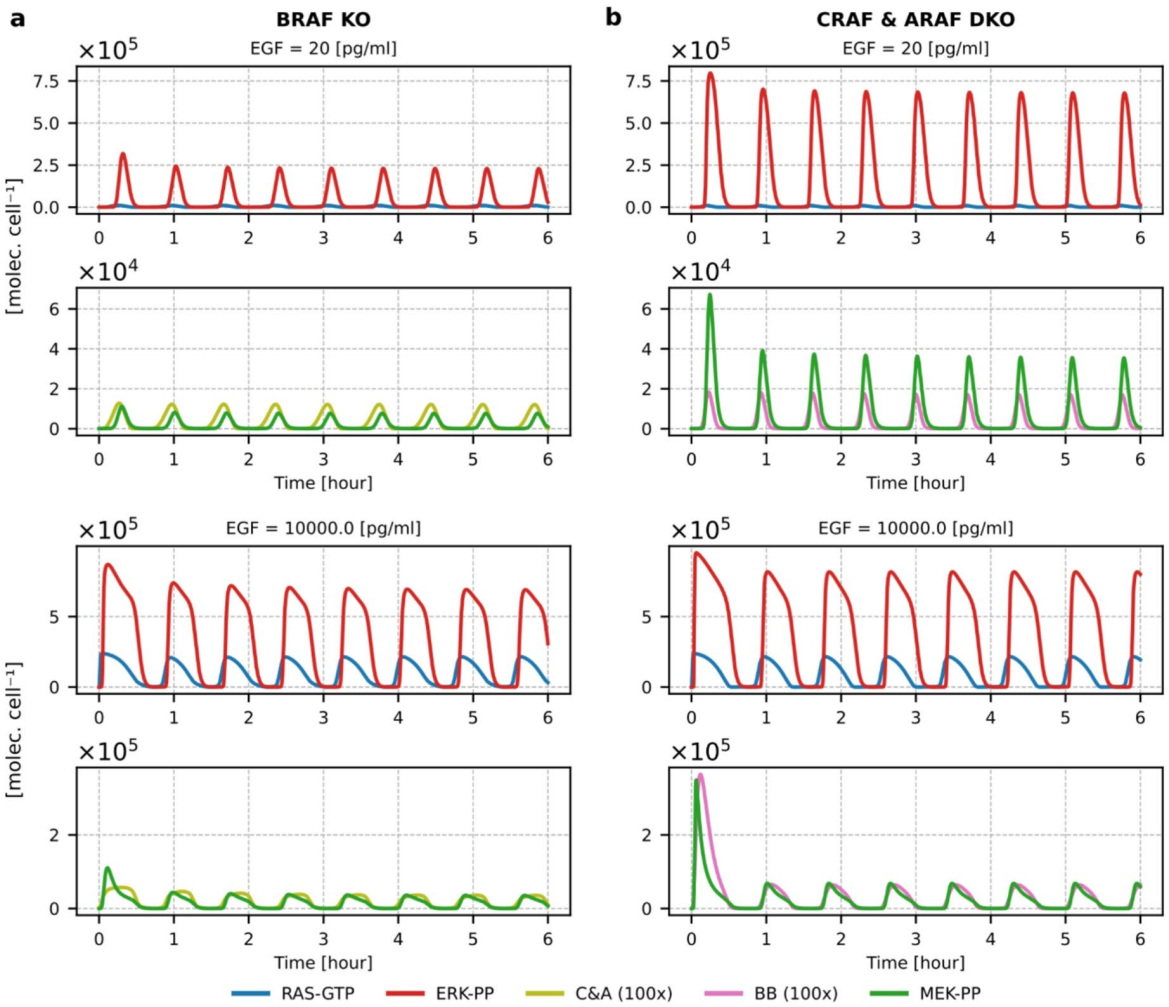
Relaxation oscillations in BRAF KO and ARAF & CRAF DKO cells. (**a**) Profiles of RAS-GTP, C&A dimers, MEK-PP, and ERK-PP in BRAF KO cells. (**b**) Profiles of RAS-GTP, BB dimers, MEK-PP, and ERK-PP in ARAF & CRAF DKO cells. Plots are shown for the low and high EGF concentrations; the corresponding plots for the intermediate EGF concentrations of 100 pg/ml and 1000 pg/ml are provided in Supplementary Figure S6.

The low abundance of C&A dimers (in WT cells) may suggest that in BRAF-deficient cells, there will be no ERK activation at all. However, this is not what we observe in the model (Fig. 5a and Supplementary Fig. S6a). There are three reasons for this: (1) the positive feedback coupling RAS and SOS is upstream of RAFs, and therefore RAS is activated regardless of the presence or absence of BRAF; (2) the number of dimers that do not contain BRAF (i.e., C&A dimers) is much higher in BRAF-deficient cells than in WT cells (compare Fig. 4 and Supplementary Figure S5 with Fig. 5a and Supplementary Fig. S6a), because in the latter case, there is no competition between dimers. Finally, (3) the model assumes high rates of MEK and ERK activation, so even a relatively low number of RAF dimers leads to ERK activation (for EGF concentrations above 100 pg/ml) comparable to that observed in WT cells, even if MEK activation is weaker. Only for the lowest considered EGF concentration of 20 pg/ml, the pulses of ERK activity are about three times lower than in WT cells (Fig. 5a vs. Fig. 3a). Reducing MEK and ERK activation constants would make the effect of BRAF knockout more pronounced. Nevertheless, experimental data indicate that BRAF inhibitors alone reduce ERK activation only modestly, and combinations of BRAF and MEK inhibitors must be used to inhibit ERK activity^23,62–64^. The effect of CRAF and ARAF double knockout is even weaker; for all considered EGF concentrations, ERK activity is similar to that in WT cells, while MEK activity is lower only for the weakest EGF stimulation (Fig. 5b and Fig. S6b versus Fig. 3a). Analysis of the RAF isoforms knockouts indicates positive feedback at the SOS-RAS level, and high signal amplification at MEK and ERK levels assures robustness of ERK activation, allowing RAF isoforms to be engaged in functions beyond ERK activation.

**Fig. 6:**
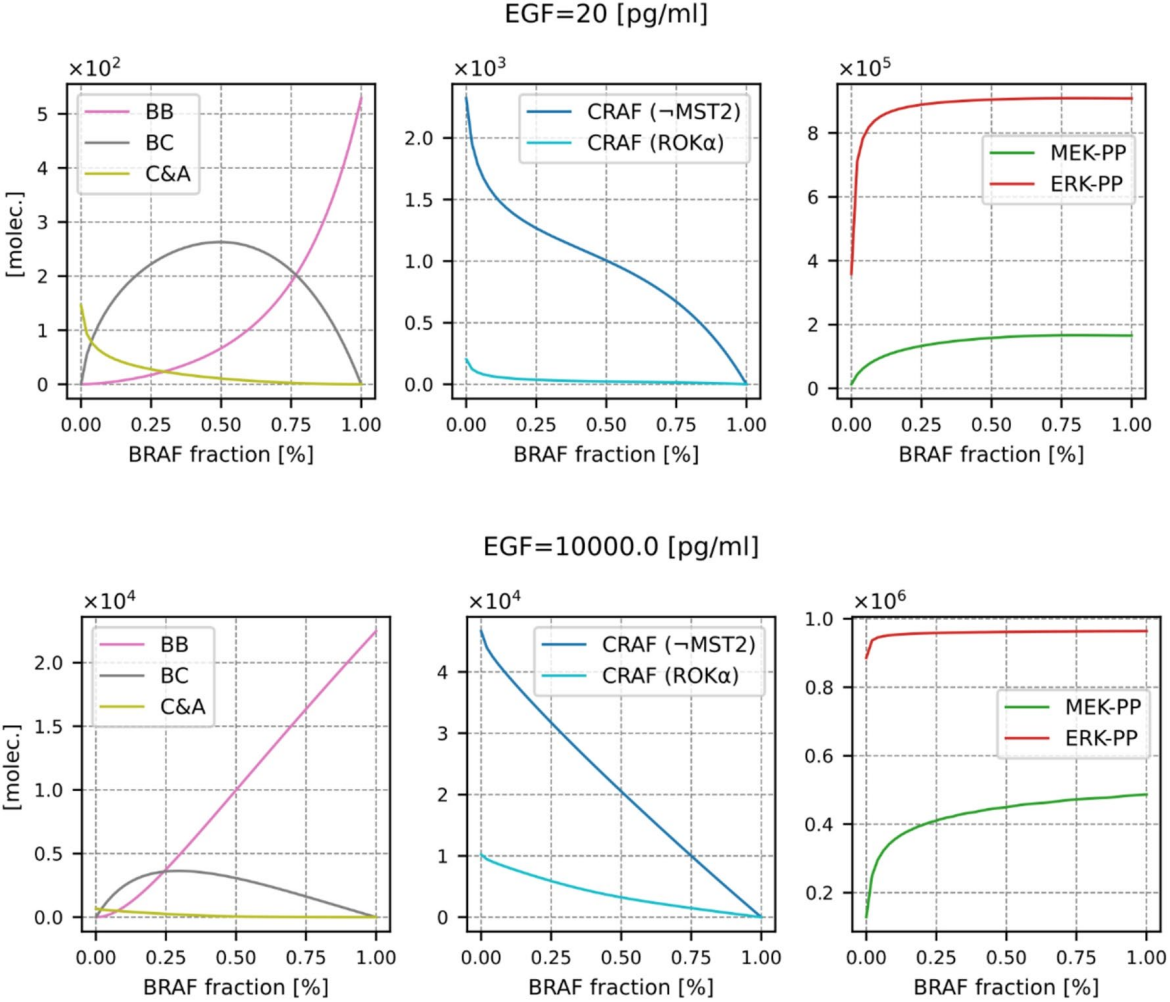
Peak abundances of the main system components as a function of the BRAF fraction. The total abundance of RAF isoforms is assumed to be constant, and the abundances of CRAF and ARAF are equal. The analysis is performed for low and high EGF concentrations (20 pg/ml and 10 000 pg/ml), the analogous plots for intermediate EGF concentrations of 100 pg/ml and 1000 pg/ml are shown in Supplementary Fig. S7.

In Fig. 6 and Supplementary Fig. S7, we analyze peak levels of the main pathway components as a function of the BRAF proportion to observe that MEK and ERK activity weakly depend on the BRAF fraction (provided that this fraction is higher than 10%) - the strongest dependence is observed at the lowest EGF concentration. Obviously, the abundances of different RAF dimers strongly depend on the BRAF proportion, with the highest level of BC heterodimers observed for a BRAF fraction between 25% and 50% (depending on EGF concentration). The total level of all CRAF-containing dimers is reduced in BRAF-deficient cells, in agreement with Freeman et al., who showed that in BRAF KO cells, CRAF catalytic activity is reduced by 90%^65^. The levels of CRAF competent to bind ROKα, and CRAF incompetent to bind MST2, decrease as the fraction of BRAF increases.

### Gradual and switch-like activation observed at subsequent tiers of the MAPK pathway

**Fig. 7:**
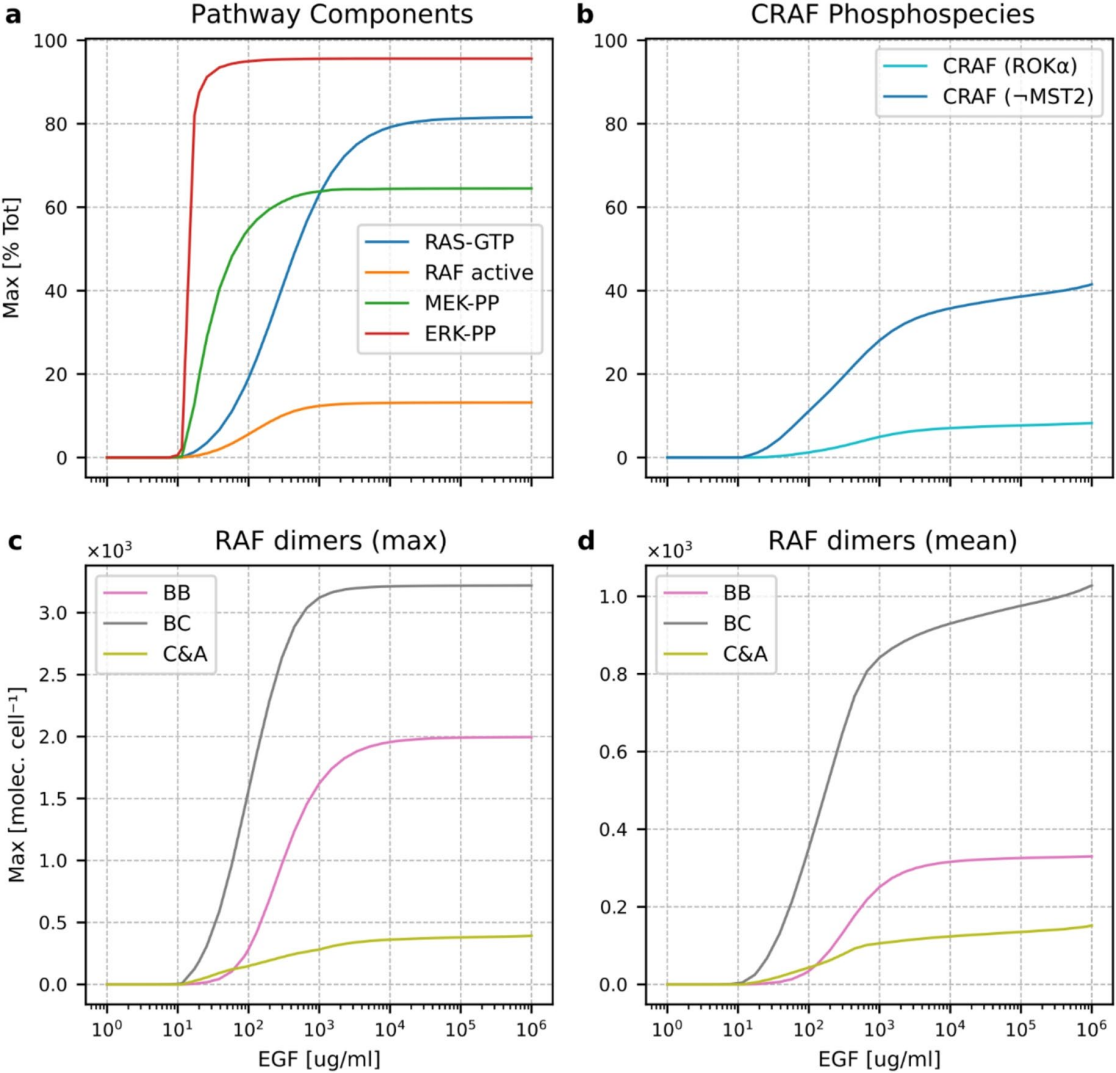
Magnitude of activation of MAPK cascade components as a function of EGF concentration. (**a**) The peak value of RAS-GTP, active RAF, MEK-PP, and ERK-PP, as a fraction of the total protein level. (**b**) The peak value of CRAF (as a fraction of total CRAF protein level) in the state in which it can form complexes with ROKα and in the state in which it cannot form complexes with MST2. (**c**) The peak value of BB, BC, and C&A dimers. (**d**) The mean value over the first 10 h of EGF stimulation of BB, BC, and C&A dimers.

The presence of a bistable switch that allows for RAS activation on a portion of the membrane and strong signal amplification at the MEK and ERK levels causes ERK activation to have a switch-like character (with the fitted Hill coefficient, *n*_*H*_, equal to 12.1 and the EC90/EC10 ratio equal to 1.6, while RAS and RAF activation can be characterized as gradual with *n*_*H*_ = 1.1, EC90/EC10 = 60 for RAS and *n*_*H*_ = 1.5, EC90/EC10 = 22 for RAF, respectively (Fig. 7a; Table 1). The Hill coefficients have been calculated by fitting the Hill functions to the activation profiles. Of note, the peak and average levels of BC and C&A dimers increase gradually with EGF concentration (Fig. 7c and d, which allow the interaction of CRAF with ROKα and MST2 to change gradually with EGF concentration (Fig. 7b). The level of CRAF competent to bind ROKα increases nearly linearly with *n*_*H*_ = 0.9 in a broad range of EGF (EC90/EC10 = 465); the level of CRAF incompetent to bind MST2 increases similarly (Table 1). These model features are in line with the experimental data, which show a gradual CRAF activation and a switch-like ERK activation in response to serum (FCS) stimulation^19^. Also, the abundance of CRAF and ROKα complexes grows gradually with the stimulation level^20,26^. The gradual RAS/RAF activation and switch-like activation of MEK/ERK are also observed in BRAF KO and CRAF&ARAF DKO cells (Supplementary Figs. S8 and S9). It should be noticed that, because both of the knockout variants preclude the formation of BC and BA heterodimers, the proportion of active RAF is much lower than in WT cells.

**Table 1:** Parameters characterizing dose response curves (peak values with respect to EGF concentration) at five tiers of the MAPK pathway. Hill coefficients and EC10, EC50, EC90 values in pg/ml were obtained by fitting to the Hill function.

Observable	Hill coeff.	EC10	EC50	EC90	EC90/EC10
EGFR–SOS	1.9	78	277	853	10.9
RAS–GTP	1.1	45	301	2724	60
RAF active	1.5	30	127	654	22
MEK-PP	2.1	14.3	30	159	11.1
ERK-PP	12.1	12.0	14.7	19.3	1.6
CRAF ROKα-competent	0.9	70	638	32,340	465
CRAF MST2-incompetent	0.9	37	373	32,851	898
BB dimers	1.4	81	312	2006	25
BC dimers	1.5	26	105	460	17.5

### Dual role of 14-3-3 in RAF isoforms activation

The 14-3-3 dimers play opposing roles in RAF activation. On one hand, they stabilize the closed, autoinhibitory conformation of RAF monomers; when the kinase domain binds the autoinhibitory N-terminal part of the protein, 14-3-3 can simultaneously bind its N- and C-terminal phosphosites. Furthermore, 14-3-3 binding to the N-terminal phosphosite interferes with stable binding to RAS, inhibiting recruitment, dimerization, and activation. On the other hand, 14-3-3 stabilizes RAF dimers by crosslinking the protomers’ C-terminal domains, which promotes RAF activity. As discussed before (within the model), this stabilization effect of 14-3-3 takes place only for dimers that contain BRAF. The picture becomes even more complicated as all three RAF isoforms compete both for binding RAS and for binding to- other RAF isoforms.

**Fig. 8:**
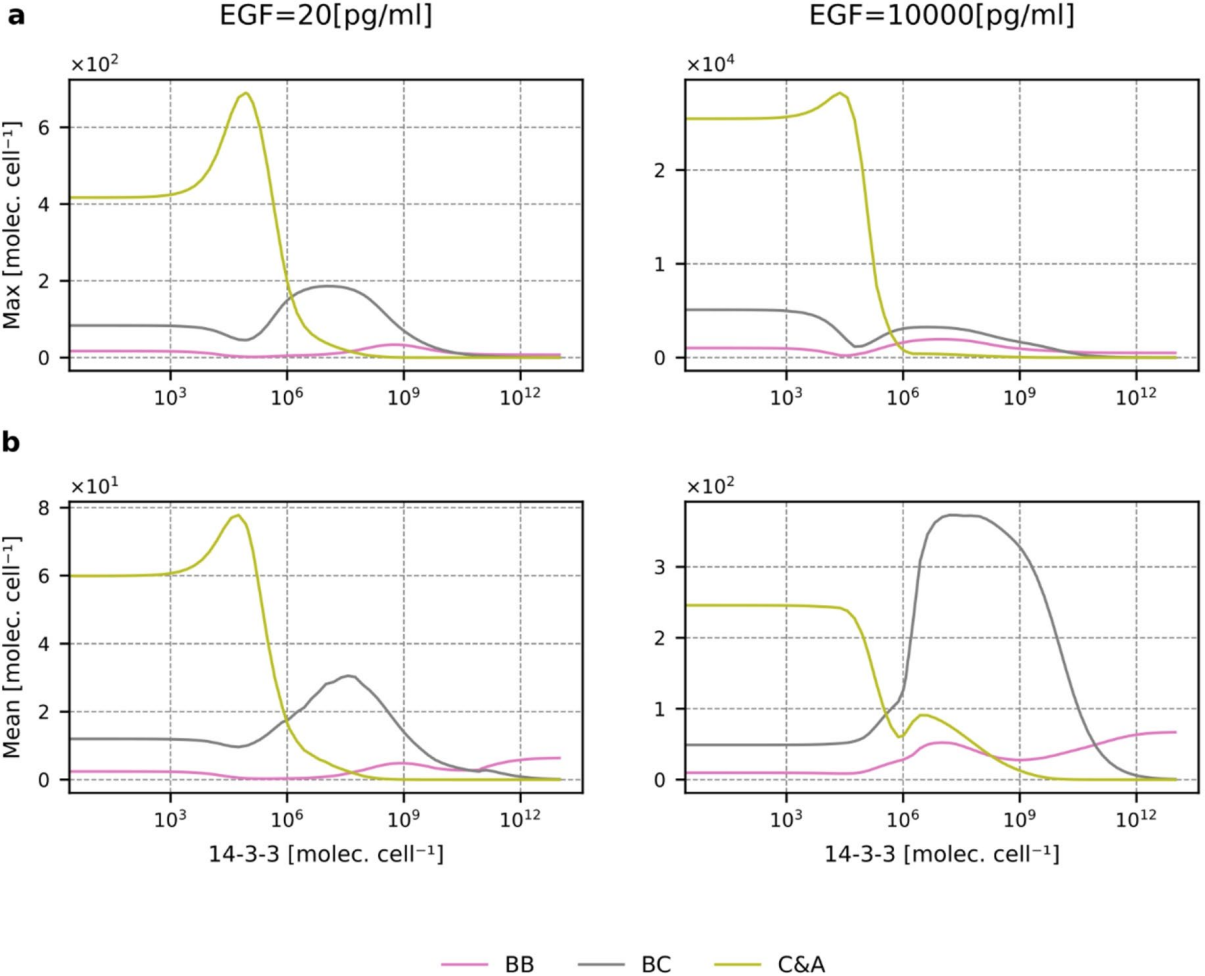
Abundance of RAF isoform dimers as a function of the 14-3-3 level. (**a**) The peak value of BB, BC, and C&A dimers as a function of the 14-3-3 level for the low and high EGF concentrations (20 and 10000 pg/ml). (**b**) The average value of BB, BC, and C&A dimers (in the first 10 h of EGF stimulation) as a function of the 14-3-3 level for the low and high EGF concentrations (20 and 10000 pg/ml). The plots corresponding to those in (**a**) and (**b**), but for the intermediate EGF concentrations of 100 and 1000 pg/ml, are shown in Supplementary Figure S11.

First, let us note that 14-3-3 is required for system oscillations. When the 14-3-3 level is low, equal to 105 (i.e., 100 times lower than nominal), we observe only a single peak of active RAF dimers (Supplementary Fig. S10). As one could expect, the opposing effects of 14-3-3 on RAF activation and 14-3-3-level-dependent competition between RAF monomers lead to complex and highly nonlinear dependence of the levels of RAF dimers on 14-3-3 abundance. In Fig. 8 and Supplementary Fig. S11, we show the dependence of the peak and average levels of RAF dimers on the 14-3-3 level (for several EGF concentrations). First, let us notice that when the level of 14-3-3 is small (so its effect on RAFs is negligible), the peak and average values of BB, BC, and C&A RAF dimers remain (regardless of the EGF concentration) in proportion 2:10:50, which reflects the relative abundances of the RAF isoforms. This is because the only differences between ARAF, BRAF, and CRAF isoforms within our model are associated with their (complex) interactions with 14-3-3.

**Fig. 9:**
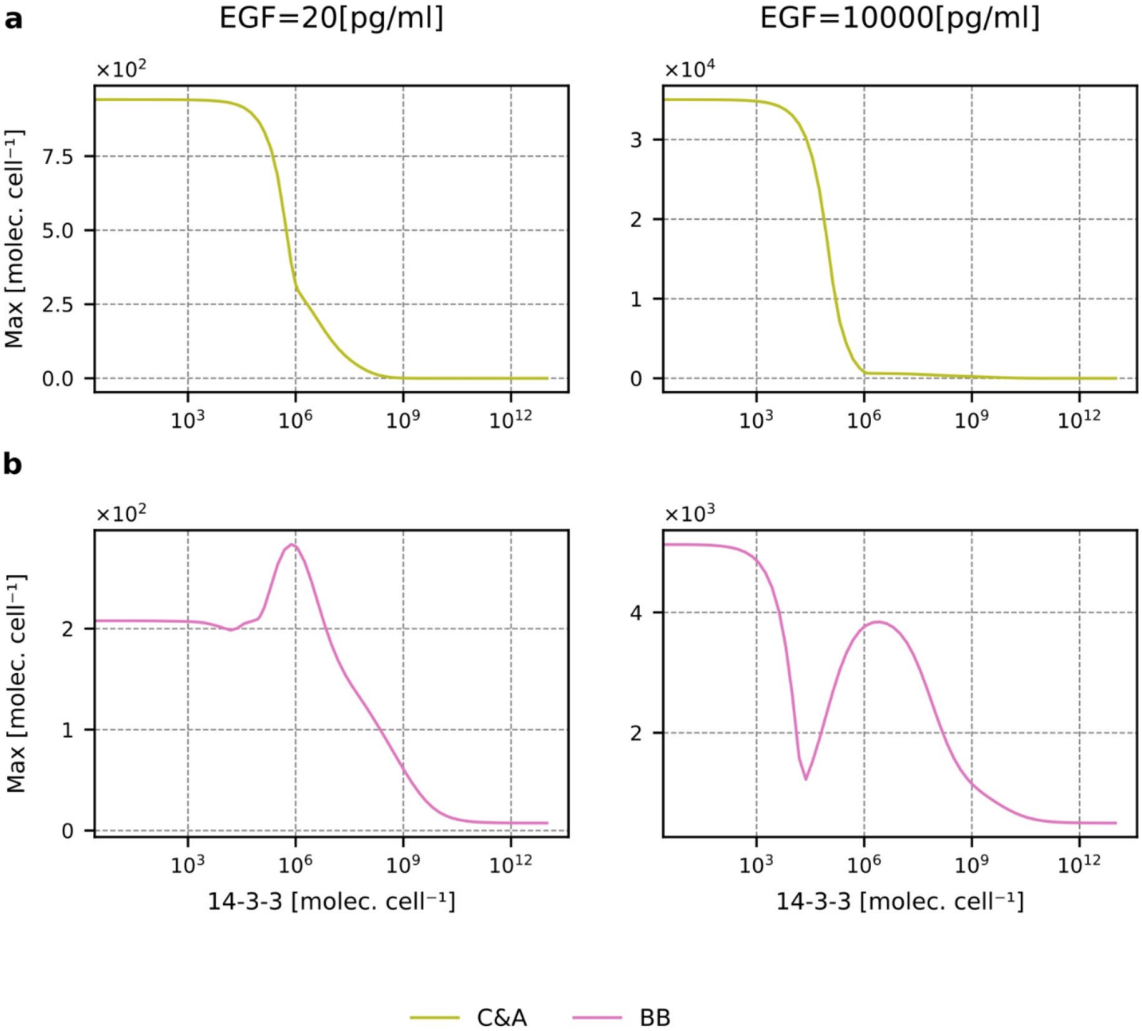
Formation of RAF isoform dimers as a function of 14-3-3 level for BRAF KO and CRAF & ARAF DKO cells. (**a**) The peak value of C&A dimers in BRAF KO cells as a function of the 14-3-3 level for the low and high EGF concentrations (20 and 10 000 pg/ml). (**b**) The peak value of BB dimers in CRAF & ARAF DKO cells as a function of the 14-3-3 level for the low and high EGF concentrations (20 and 10 000 pg/ml). The plots corresponding to those in (**a**) and (**b**), but for the intermediate EGF concentrations of 100 and 1000 pg/ml, are shown in Supplementary Figure S10.

An increase in 14-3-3 is associated with a small increase, followed by a sharp decrease in C&A dimers (Fig. 8 and Supplementary Fig. S11). In BRAF-deficient cells (Fig. 9a and Supplementary Fig. S12a), regardless of EGF concentration, the peak level of C&A dimers decreases monotonically with the number of 14-3-3 molecules (which stabilize CRAF and ARAF monomers in the closed conformation). This indicates that the initial increase in C&A dimers results from competition with BRAF for binding RAS-GTP. This explains why the relative growth of the peak level of C&A dimers is higher for low EGF concentrations when the available pool of RAS-GTP is small. BC heterodimers are most abundant for physiological 14-3-3 concentrations (10^7^−10^8^ molecules per cell), while for the low and very high 14-3-3 levels their abundance is lower because, respectively, dimers are not stabilized by 14-3-3, or CRAF and BRAF are captured in their closed conformations, so the dimers are not formed.

The regulation of BRAF homodimers is most complex, as visualized in ARAF and CRAF DKO cells for EGF concentrations higher than 100 pg/ml (Fig. 9b and Supplementary Fig. S12b). We may observe that an increase in the14-3-3 level first leads to a decrease of the BB homodimer level due to inhibition of BRAF monomers, then to an increase due to homodimers’ stabilization, and then again to a decrease. Let us notice that the strength of dimer stabilization only initially increases with the level of 14-3-3 – for a very high level of 14-3-3, each of the BRAF protomers can be loaded with its ‘own’ 14-3-3, which precludes dimer stabilization. The same effect is not observed in the case of monomer stabilization, as a single BRAF molecule cannot be bound to two 14-3-3 molecules, and thus inhibitory effect increase with 14-3-3 level and prevails at high 14-3-3 concentrations, reducing the number of BRAF homodimers.

## Discussion

We have constructed a rule-based model of the MAPK/ERK pathway to investigate the system’s kinetics. The focus was on regulatory feedback loops, RAF isoforms, and their interactions with 14-3-3 proteins. Instead of fitting to particular cell-line responses, we explored the parameter space and RAF and MEK isoform knockouts to investigate the repertoire of responses to growth factor stimulation.

The model accounts for one positive and three negative feedback loops. The positive feedback loop involves SOS and RAS, and is responsible for the system’s bistability observed upon silencing the negative feedback loops. All three negative feedback loops emanate from ERK but have distinct roles.

1. The feedback loop involving SOS is dynamically most important. It encompasses the positive feedback loop, which allows it to frustrate the system’s bistability and induce relaxation oscillations. Consequently, pulsatile and constant-level modes of growth factor stimulation are converted into pulsatile ERK responses.
2. The feedback loop involving ERK phosphorylation of RAF at DIF domain disrupts RAF dimers, terminating MEK signaling but enabling further interaction of CRAF monomers with ROKα, promoting cell motility, and with ASK1, inhibiting apoptosis.
3. The proximal ERK-MEK feedback loop functions to shape ERK activity pulses. Its strength increases with the MEK1/MEK2 ratio.

To reconcile nearly all-or-nothing ERK activation with a gradual activation of RAF kinases, we proposed that at low and intermediate EGF concentrations, growth factor receptors are activated on a portion of the cellular membrane. Consequently, the positive feedback between SOS and RAS induces switching of RAS-GDP to RAS-GTP locally on the cell membrane. Activation of a small proportion of cellular RAS leads to activation of a small proportion of RAF kinases. This is, however, sufficient to trigger nearly full ERK activation because of the ultrasensitivity associated with MEK and ERK double phosphorylation. The proposed mechanism allows the MAPK pathway to simultaneously mediate graded responses at the RAS and RAF level and switch-like responses at the ERK level. Ultrasensitive, all-or-nothing ERK activation enables temporal pathway inactivation in response to both weak and strong signals.

We demonstrated that the different activation patterns of the RAF isoforms, and thus RAF dimers, can be attributed to their distinct regulation by 14-3-3. 14-3-3 dimers stabilize both the closed, inactive conformation of RAF monomers and the catalytically active conformation of RAF dimers containing BRAF. The specificity of BRAF is caused by the fact that its primary 14-3-3 binding site is on its C-terminal, and therefore (in contrast to CRAF and ARAF) it may bind RAS-GTP with the N-terminal site, without dissociating from 14-3-3. Of note, the inability of CRAF to bind 14-3-3 solely by its C-terminal residues was demonstrated earlier by Varga et al. by showing the absence of CRAF-14-3-3 complexes in CRAF-S259A mutants^26^, as well as earlier by Fischer et al. 2009 who showed that the substitution S259A in CRAF or S214A in ARAF strongly reduced the phosphorylation of CRAF and ARAF on the C-terminal sites, Ser621 and Ser582 (suggesting dissociation of 14-3-3 from these sites)^14^. Recently, however, Jang et al. showed in a Cryo-EM study the presence of CRAF/MEK1/14-3-3 complexes, with 14-3-3 bound to open CRAF at Ser621 and stabilized by glancing contact with the C-lobe of MEK1^17^. In light of this study, the assumption that CRAF and ARAF’s inability to bind 14-3-3 solely by C-terminal residues should be considered a model idealization.

Consequently, following the model assumptions, BRAF may donate 14-3-3 to stabilize BC and BA heterodimers. BB homodimers can also be stabilized after 14-3-3 dissociates from one of the monomers, allowing the remaining one to crosslink the dimer. CRAF and ARAF may bind RAS-GTP only after 14-3-3 dissociates, and thus, RAF dimers that do not contain BRAF are not stabilized by 14-3-3, and their abundance is much lower. Nevertheless, the model predicts that although in WT cells BRAF-containing dimers dominate, the effect of BRAF knockout on ERK activity is relatively weak. This is because (1) SOS-RAS positive switch turns on independently of RAF signaling, (2) BRAF deficiency leads to an increase in C&A dimers, and (3) ultrasensitivity at MEK and ERK level allows for nearly full ERK activation at low levels of active RAFs.

Because14-3-3 stabilizes both inactive RAF monomers and active RAF dimers (containing BRAF) causes the abundance of specific RAF dimers to be a non-monotonic function of the 14-3-3 level. The complexity of the picture is further increased by competition between RAF isoforms for RAS-GTP (important in the case of low EGF concentrations). Consequently, the model predicts qualitatively different dependencies of BC, BB, and C&A dimers on the level of 14-3-3. For the nominal level of 14-3-3, the peak value (and the average level over the first 10 h of EGF stimulation) of BC dimers is higher than that of BB and C&A dimers.

The relative abundance of RAF dimers and engaged RAF monomers is not important for ERK kinase activity but becomes crucial when the interaction of CRAF and ARAF with other proteins is considered. Engagement of CRAF and ARAF in RAF dimers releases MST2, allowing it to participate in the proapoptotic Hippo pathway^19^. The model indicates that the CRAF and ARAF fraction capable of binding MST2 decreases gradually with EGF concentration, suggesting that strong, long-lasting EGF signals can be proapoptotic, depending on the status of other pathways, such as PI3K/AKT. CRAF is released from BC, AC, and CC dimers in the open conformation, in which it can bind ROKα, promoting cell motility and coordinating it with proliferation^26^.

In summary, the constructed model indicates that the presence of RAF and MEK isoforms, the distinct interactions of RAF isoforms with 14-3-3, and the regulatory features of the MAPK/ERK pathway allow for a broad repertoire of dynamic responses to growth factor stimulation. This implies that different cell types, having specific proportions of signaling proteins, may exhibit qualitatively different responses, employing the MAPK/ERK pathway for diverse physiological functions.

## Methods

### Rule-based modeling

The model was implemented in a rule-based paradigm, using BioNetGen language (BNGL) within the BioNetGen environment (the code provided as Supplementary Code S1)^45,46,66,67^. BNGL models are formulated by enumerating rules, which encode possible reaction patterns. A set of rules specifying a model is then automatically processed to generate the reaction network and the corresponding system of coupled ODEs; the system is assumed to follow mass-action kinetics. The systems of ODEs were numerically integrated using solvers provided with BNGL (CVODE) at default settings.

### Model description

The model follows the established basic growth factor signal transduction scheme in the MAPK/ERK cascade. Briefly, EGF binds and activates the EGFR receptor, which can then bind SOS. EGFR-bound SOS subsequently activates RAS. Activated RAS binds and activates RAF isoforms. Activated RAF subsequently phosphorylates and activates MEK1/2, which do the same to ERK1/2. ERK1/2 phosphorylates SOS, RAF isoforms, and MEK1, disrupting their activity and forming negative feedback loops. The positive feedback between SOS and RAS arises because RAS-GTP allosterically binds to SOS, promoting the conversion of RAS-GDP to RAS-GTP.

The model was implemented in BNGL due to its significant inherent combinatorial complexity. It comprises 11 proteins: EGFR, SOS, RasGAP, RAS, the BRAF/CRAF/ARAF isoforms, 14-3-3, the MEK1 and MEK2 isoforms, and ERK. It has been specified in BNGL by defining 122 reaction rules and parameters (provided in Supplementary Text S1), generating a network of 9088 chemical reactions and 609 molecular species represented by the corresponding system of 609 coupled ODEs.

### The RAF isoforms

The model accounts for all three known vertebrate RAF isoforms: ARAF/BRAF/CRAF . Their structure and regulation have been implemented in significant mechanistic detail. Each isoform follows the structure of a generic RAF protein, featuring the N- and C-terminal 14-3-3 binding sites, the N-terminal acidic domain (NtA), and the dimerization interface DIF. The RAF isoforms are known to possess the inactive “closed” and active “open” conformations. In the “closed” conformation, the C-terminal catalytic domain-containing segment of the protein is occluded by the N-terminal autoinhibitory segment; this conformation is further stabilized by the intramolecular crosslinking by a 14-3-3 protein bound at the N-terminal and C-terminal phosphosites. In contrast, in the active “open” conformation, the interaction between the N- and C-terminal segments is disrupted, allowing for the interaction between the catalytic domain and substrates. This conformation is likely stabilized by phosphorylation of the NtA domain, and this phosphorylation is used as a proxy for the “open” active RAF. The RAF isoforms share the same activation scenario:

1. Dimerized RAS-GTP recruits RAF protein monomers. For this interaction to occur, the N-terminal 14-3-3 binding site must be unoccupied.
2. The two RAF proteins bound to the RAS-GTP dimer subsequently dimerize. The RAF dimers containing BRAF are stabilized by the intramolecular crosslinking via the 14-3-3 bound to the C-terminal site.
3. Dimerization triggers the autophosphorylation of the NtA domains of the constituent RAF protomers. A dimerized RAF molecule phosphorylated at the NtA domain is considered catalytically active.
4. The RAF dimer detaches itself from the RAS-GTP dimer.
5. ERK phosphorylates the DIF domain of the RAF protein, triggering the breakdown of the dimers and terminating signaling.

While the RAF isoforms share the same overall structure, there are discernible differences between them in terms of activity and regulation, hinting at specialized functions. A significant difference between them is that in BRAF, the high-affinity 14-3-3 binding site is the C-terminal one, unlike the N-terminal one in ARAF and CRAF. As a consequence, the ARAF and CRAF proteins bound to RAS-GTP are devoid of 14-3-3, while BRAF can maintain its interaction with 14-3-3 via its C-terminal site. This favors the stability of BRAF heterodimers with other isoforms, as BRAF can act as a donor of 14-3-3 to the C-terminal binding site of the other isoform within the dimer, which is likely to be unoccupied and thus lends itself to crosslinking and dimer stabilization. In the case of BRAF homodimers, however, the simultaneous occupancy of both C-terminal binding sites by two distinct 14-3-3 proteins can interfere with crosslinking and stabilization. ARAF and CRAF homodimers and CRAF-ARAF heterodimers are not crosslinked by 14-3-3, which effectively reduces their stability. This explains the relative abundance of BRAF-containing heterodimers, which accounts for the observation that BRAF heterodimers (BC in particular) are the main activators of MEK1/2.

A recent Cryo-EM study indicated that BRAF can bind to RAS even in its closed conformation, implying the dissociation of 14-3-3 from the N-terminal site after binding, allowing BRAF to assume the open conformation^16^. In the model, this intermediate step is omitted, and we assume that all RAF isoforms bind RAS with 14-3-3 dissociated from their N-terminal sites. Another Cryo-EM study showed the existence of MEK1-CRAF-14-3-3 complexes with open CRAF bound solely by Ser621 to the 14-3-3 dimer^17^. In these complexes, 14-3-3 is stabilized by glancing contact with the C-lobe of MEK1 (residues 238–241). Nevertheless, one may not exclude the possibility that there is some fraction of CRAF bound to 14-3-3 solely by the C-terminal residue. Cautiously, our model assumption that CRAF and ARAF release 14-3-3 prior to RAS binding must be considered an idealization.

### Nested feedback architecture

It has been experimentally observed that the RAF/ERK cascade is capable of producing ERK-PP pulses with a nearly EGF concentration-independent amplitude but an EGF concentration-dependent period. This suggests frequency-based, rather than typically assumed amplitude-based, encoding of the signal strength. We previously demonstrated that the RAF/ERK cascade could exhibit this behavior if it were capable of relaxation oscillations^40^. Relaxation oscillations involve periodic and abrupt alternations between two stable equilibrium states, resulting in non-sinusoidal repetitive patterns such as spikes. This behavior emerges from the activity of two opposing but coupled feedback processes with significantly differing time scales, where the slow-acting negative feedback loop encloses the fast-acting positive feedback loop. We previously identified such a motif in the structure of the RAF/ERK cascade, demonstrated that it can produce the observed frequency-based encoding, and now incorporate it into the current model.

The outer slow-acting negative loop is based on the negative feedback phosphorylation of SOS by ERK-PP. SOS contains four sites targeted by ERK, and phosphorylation of any of them precludes its binding to activated EGFR receptors and membrane recruitment. This forms the longest and correspondingly the slowest feedback loop in the RAF/ERK cascade. SOS is also the target of the inner fast-acting positive feedback loop. SOS undergoes positive regulation by its activated substrate RAS-GTP. In particular, RAS-GTP can bind SOS via a separate domain (REM) and allosterically increase its GEF activity, which establishes a positive feedback loop. This feedback loop is fast due to its short range. In summary, SOS is a nexus of a long, slow-acting negative feedback loop and the enclosed short, fast-acting positive feedback loop, which produce relaxation oscillations.

### Signaling scenario

In unstimulated cells, RAS is in the GDP-bound monomeric state on the plasma membrane, while SOS is in the cytoplasm in its inactive form and unphosphorylated on its ERK feedback sites. The BRAF, CRAF, and ARAF isoform molecules are in the inactive “closed” conformation and are phosphorylated on their N-terminal (BRAF – Ser365, CRAF – Ser259, ARAF – Ser214) and C-terminal (BRAF – Ser729, CRAF – Ser621, ARAF – Ser582) 14-3-3 binding sites, which enables further “locking” of the conformation by 14-3-3 crosslinking. They are also unphosphorylated on their respective NtA domains (CRAF – Ser338, ARAF – Ser299). In contrast, BRAF is assumed to be constitutively phosphorylated on its NtA site (Ser446). It is assumed that, in contrast to CRAF and ARAF, BRAF may be crosslinked by 14-3-3 when phosphorylated at Ser446. All RAF isoforms are unphosphorylated on their DIF region. MEK and ERK are inactive, unphosphorylated on their activation loops and feedback sites.

Upon stimulation with the ligand, EGFR becomes activated and recruits SOS to the membrane. The rate of EGFR activation corresponds to the concentration of EGF scaled by the fraction of the membrane undergoing activation, *f* = *EGF*/(*EGF +* M_EGF_), with M_EGF_ = 300 pg/ml, i.e., we assume that at low EGF concentration only part of the membrane becomes active. SOS subsequently recruits RAS-GDP via its GEF domain and induces RAS to exchange GDP for GTP, which leads to its activation and dimerization. SOS can also independently bind RAS-GTP via the REM domain, which allosterically upregulates its GEF activity. A RAS-GTP monomer can be recruited and deactivated by RasGAP by inducing a transition from RAS-GTP back to RAS-GDP.

Dimerized RAS-GTP recruits two RAF monomers; to be competent for RAS recruitment, RAF isoform molecules have to be free of 14-3-3 at their N-terminal binding sites, which enables interaction with RAS. The bound RAF proteins can subsequently dimerize on the RAS-GTP dimer platform via their DIFs. The dimers containing BRAF may be stabilized by 14-3-3 crosslinking via the C-terminal 14-3-3 binding sites. RAF dimers subsequently detach from the RAS dimers. RAF dimers are considered catalytically active and phosphorylate MEK1/2 on their activation loops. MEK1/2 consequently activate ERK by phosphorylating its activation loop.

Phosphorylated ERK can disrupt the activity of upstream components, forming negative feedback loops. We have accounted for the following:

1. ERK phosphorylates four sites on SOS, each individually sufficient to prevent the interaction between EGFR and SOS. This loop is part of the relaxation oscillation motif.
2. ERK phosphorylates RAF monomers and protomers on the DIF region, blocking dimerization and disrupting existing dimers.
3. ERK phosphorylates MEK1 at Thr292, which (by recruiting an implicit phosphatase or phosphatase-containing complex) facilitates dephosphorylation of the MEK activation loop.

The negative feedback to RAF dimers plays a relevant role, independent of pathway deactivation. Specifically, CRAF and ARAF monomers produced from disrupted dimers retain phosphorylation of their NtA regions; this keeps them in the “open” conformation and enables interaction of CRAF with ROKα.

## Supplementary Information

Below is the link to the electronic supplementary material.

Supplementary Material 1

Supplementary Material 2

## Data Availability

Data supporting this study are included within the article and supporting materials.
