# Supplementary material for: A computational rule-based model of MAPK/ERK system regulation
Source: Sci Rep. 2026 Mar 21;16:14437. doi: 10.1038/s41598-026-44353-3 (PMC13149509; doi:10.1038/s41598-026-44353-3)
Supplement: Supplementary file 2 — Supplementary Material 2 [file 41598_2026_44353_MOESM2_ESM.pdf]

Supplementary Text S1
(with Supplementary Figures S1-S12)

A computational rule-based model of MAPK/ERK system regulation

Paweł Kocieniewski[1,✉] , Tomasz Lipniacki[1,✉]

[1] Institute of Fundamental Technological Research, Polish Academy of Sciences, Warsaw, Poland

✉Corresponding authors: pkocien@ippt.pan.pl (P.K.), tlipnia@ippt.pan.pl (T.L.)

Computational Model

Species definitions

EGFR(egf~I~A, sos) – EGFR, EGF receptor

- **egf:** indicates the inactive (I) or active (A) state of the receptor
- **sos:** the SOS binding domain, active receptor can bind SOS

SOS(egfr,rem,S~P0~P1~P2~P3~P4) – SOS, a guanine nucleotide exchange factor (GEF), activates RAS-GDP by inducing nucleotide exchange from GDP to GTP and producing RAS-GTP

- **egfr**: the EGFR-binding site
- **rem**: a secondary RAS binding domain; when occupied by RAS it allosterically enhances the GEF activity of SOS
- **S**: a flag to indicate the number of phosphorylated inactivating sites, SOS possesses four such sites, which are subject to distributive and independent feedback phosphorylation by ERK1/2; phosphorylation of any of them precludes SOS-EGFR binding.

RasGAP(ras) – RAS GTP-ase Activating Protein, which inactivates RAS-GTP by inducing the hydrolysis of GTP to GDP:

- **ras:** the RAS binding domain

RAS(sos,dim,raf,nt~GDP~GTP) – RAS, a small GTPase, binds and recruits RAF isoforms to the plasma membrane and initiates their activation:

- **sos**: the SOS binding site
- **dim**: the dimerization interface
- **raf**: the RAF binding site
- **nt**: a flag to indicate the activation status of RAS. RAS can be loaded either with GDP (**nt~GDP** – considered inactive) or GTP (**nt~GTP** – considered active). GDP and GTP are not explicitly represented.

RAF(iso~B~C~A,ras,N_ftt~P0~P1,dif~P0~P1,NtA~P0~P1,C_ftt~P0~P1) – a RAF kinase with BRAF, CRAF, and ARAF isoforms

Due to the regulatory and structural similarities between RAF isoforms we introduced a generalized RAF isoform species. When an isoform deviates in its regulation from the assumed baseline, it is explicitly identified in the corresponding rules.

- **iso:** specifies the identity of the isoform (B/C/A) if required
- **ras:** the RAS binding site, collapses both the RBD and CRD domains
- **N_ftt:** the N-terminal 14-3-3 binding site; ARAF – Ser^{214} , CRAF – Ser^{259} BRAF – Ser^{365}
- **dif:** the dimerization interface/domain
- **NtA:** the N-terminal acidic domain whose phosphorylation promotes the active conformation and protein-protein interactions; ARAF – Ser^{299} , CRAF – Ser^{338}, BRAF – Ser^{446}
- **C_ftt:** the C-terminal 14-3-3 binding site, ARAF – Ser^{582} ,CRAF – Ser^{621}, BRAF – Ser^{729}

MEK(iso~mek1~mek2,dim,NFS~P0~P1,al~P0~P1~P2) – MEK kinase with MEK1 and MEK2 isoforms, ERK-activating kinases

- **iso:** the identity of the isoform (MEK1 or MEK2)
- **dim:** the dimerization interface
- **NFS:** the ERK negative feedback site. When MEK is phosphorylated at this site it is rapidly dephosphorylated on its activation loop (**al**). Within a dimer, the phosphorylation of one protomer at this site confers the rapid dephosphorylation of the activation loop of the second protomer. This site is functional only in MEK1 (T292)
- **al:** the activation loop, biphosphorylated (**al~P2**) MEK1/2 is catalytically activated

ERK(al~P0~P1~P2) – ERK, the last (output) kinase in the canonical ERK cascade, mediates negative feedback to SOS, A/B/CRAF, and MEK

- **al**: the activation loop, when biphosphorylated (**al~P2**) ERK is catalytically activated

FTT(raf,dim,iso~A~B) – 14-3-3 protein, binds A/B/CRAF

- **raf:** a RAF isoform binding site. 14-3-3 can bind ARAF on $pSer^{214}$ and $pSer^{582}$, BRAF on $pSer^{356}$ and $pSer^{729}$, and CRAF on $pSer^{259}$ and $pSer^{621}$
- **dim:** the dimerization interface with other 14-3-3 monomers
- **iso:** specifies the minimal set of two distinct isoforms to account for homo- and heterodimeric nature of 14-3-3 proteins

RAF isoforms binding sites

**Isoform**	**N-terminal 14-3-3 binding site**	**C-terminal 14-3-3 binding site**	**NtA domain**	**DIF negative feedback sites**
ARAF	Ser214	Ser582	Ser299	Not mapped
BRAF	Ser365	Ser729	Ser446	Thr401,Ser750, Tyr753
CRAF	Ser259	Ser621	Ser338	Ser289/296/301

Parameters

1. Molecular Species

Protein Concentrations (*Homo sapiens*)				
**Parameter**	**Model** $[molec \times cell^{-1}]$	**PaxDB - Range (whole organism)** $[molec \times cell^{-1}]$	**PaxDB - Integrated (whole organism)** $[molec \times cell^{-1}]$	**Description**
$EGFR\_tot$	6×10^4	$(7.15 \times 10^4,\ 5.16 \times 10^5)$	1.01×10^5	EGFR receptor
SOS_tot	2×10^4	$(9.30 \times 10^3,\ 6.89 \times 10^4)$	1.42×10^4	SOS1
$RasGAP\_tot$	5×10^4	$(3.84 \times 10^4,\ 3.46 \times 10^5)$	2.55×10^5	RAS-GAP (RASA1/2, NF1)
RAS_tot	3×10^5	$(2.41 \times 10^5,\ 4.13 \times 10^6)$	9.95×10^5	RAS (K, N, H)
$BRAF\_tot$	2×10^4	$(5.00 \times 10^3, 1.38 \times 10^5)$	1.47×10^4	BRAF
$CRAF\_tot$	5×10^4	$(5.00 \times 10^3,\ 1.51 \times 10^5)$	1.43×10^4	CRAF
$ARAF\_tot$	5×10^4	$(1.80 \times 10^4,\ 1.96 \times 10^5)$	4.92×10^4	ARAF
MEK_tot	6×10^5	$(4.09 \times 10^5,\ 1.74 \times 10^6)$	8.95×10^5	MEK1/2
ERK_tot	1×10^6	$(5.77 \times 10^5,\ 3.34 \times 10^6)$	1.15×10^6	ERK1/2
FTT_tot	1×10^7	$(2.74 \times 10^8,\ 8.47 \times 10^8)$	6.10×10^8	14-3-3

The ranges of the provided protein abundances are based on 6 datasets (listed below) from ProteinAtlas[1] and The Global Proteome Machine (GPM)[2] reporting abundances for 'whole (human) organisms' included in the PaxDB Protein Abundance Database[3]. It is worth noting that there is significant variability between tissues and cell lines. The conversion from particle per million (ppm) follows the rule 1pmm=10 000 molec/cell as suggested in the PaxDB. The integrated abundances refer to the values provided by PaxDB, based on various data sources.

Protein Datasets:
H.sapiens - Whole organism (**Integrated**) - https://pax-db.org/dataset/9606/1825491670

1) H.sapiens - Whole organism, SC (Peptideatlas,aug,2014) - https://pax-db.org/dataset/9606/3848834427
2) H.sapiens - Whole organism, SC (Gpm,aug,2014) - https://pax-db.org/dataset/9606/2863052565
3) H.sapiens - Whole organism, SC (Peptideatlas,aug,2011) - https://pax-db.org/dataset/9606/1087153038

4) H.sapiens - Whole organism, SC (Peptideatlas,may,2010) - https://pax-db.org/dataset/9606/1532454152
5) H.sapiens - Whole organism, SC (Peptideatlas,march,2009) - https://pax-db.org/dataset/9606/149366043
6) H.sapiens - Whole organism, SC (Gpm,oct,2012) - https://pax-db.org/dataset/9606/3395297686

We assumed a lower abundance of 14-3-3 than reported for all 14-3-3 isoforms (in sum) to account for the multiple 14-3-3 binding partners beyond the MAPK/ERK pathway.

2. Kinetic Parameters

Quiescent state and signal-independent interactions		
**Parameter**	**Value**	**Description**
p_B_S365	$1 \times 10^{-1}\ [s^{-1}]$	Phosphorylation of BRAF-S365
q_B_S365p	$1 \times 10^{-1}\ [s^{-1}]$	Dephosphorylation of BRAF-S365p
b_FB_S729p	$3 \times 10^{-7}\ [molec\ \times s^{-1}]$	Binding of 14-3-3 to BRAF-S729p
u_FB_S729p	$3 \times 10^{-2}\ [s^{-1}]$	Dissociation of 14-3-3 from BRAF-S729p
p_NS_AC	$3 \times 10^{-1}\ [s^{-1}]$	Phosphorylation of CRAF-S259 and ARAF-S214
q_NSp_AC	$1 \times 10^{-2}\ [s^{-1}]$	Dephosphorylation of CRAF-S259p and ARAF-S214p
p_CS_AC	$1 \times 10^{-2}\ [s^{-1}]$	(Auto)Phosphorylation of CRAF-S621 and ARAF-S582
$p\_CS\_AC\_dim$	$100\ [s^{-1}]$	Dimer-induced (auto)phosphorylation of CRAF-S621 and ARAF-S582
q_CS_AC	$3 \times 10^{-1}\ [s^{-1}]$	Dephosphorylation of CRAF-S621p ARAF-S582p
b_FNp_AC	$1 \times 10^{-7}\ [molec\ \times s]^{-1}$	Binding of 14-3-3 to CRAF-S259p and ARAF-S214p
u_FNp_AC	$1\ [s^{-1}]$	Dissociation of 14-3-3 from CRAF-S259p and ARAF-S214p
b_RF_intra	$100\ [s^{-1}]$	Intramolecular crosslinking of A/B/CRAF by 14-3-3
$u_RF_$inter	$3 \times 10^{-2}\ [molec\ \times s]^{-1}$	Intramolecular decrosslinking of A/B/CRAF by 14-3-3
b_RF_inter	$100\ [s^{-1}]$	Intermolecular crosslinking of RAF dimers by 14-3-3
$u_RF_$intra	$3 \times 10^{-2}\ [molec\ \times s]^{-1}$	Intermolecular decrosslinking of RAF dimers by 14-3-3
b_MEK11	$1 \times 10^{-5}\ [molec\ \times s]^{-1}$	Homodimerization of MEK1
u_MEK11	$1 \times 10^{-3}\ [s^{-1}]$	Dissociation of MEK1 homodimers
b_MEK22	$1 \times 10^{-5}\ [molec\ \times s]^{-1}$	Homodimerization of MEK2
u_MEK22	$3 \times 10^{-2}[s^{-1}]$	Dissociation of MEK2 homodimers

b_MEK12	$1 \times 10^{-5}\ [molec\ \times s]^{-1}$	Heterodimerization of MEK1 and MEK2
u_MEK12	$1 \times 10^{-3}\ [s^{-1}]$	Dissociation of MEK1/MEK2 heterodimers

**Signal Transduction - Membrane**		
**Parameter**	**Value**	**Description**
a_EGFR	$3 \times 10^{-5} \times EGF\ [s^{-1}]$	Activation of EGFR by EGF
i_EGFR	$1 \times 10^{-2}\ [s^{-1}]$	Deactivation of EGFR
b_1	$1 \times 10^{-5}\ [molec\ \times s]^{-1}$	Binding of unphosphorylated SOS to activated EGFR
u_1a	$1 \times 10^{-2}\ [s^{-1}]$	Dissociation of unphosphorylated SOS from active EGFR
u_1b	$100\ [s^{-1}]$	Dissociation of unphosphorylated SOS from inactive EGFR
b_2a	$1 \times 10^{-6}/frac\ [molec \times s]^{-1}$	Binding of RAS-GDP to SOS(rem)
u_2a	$1\ [s^{-1}]$	Dissociation of RAS-GDP from SOS(rem)
b_2b	$b\_2a \times PF\quad [molec\ \times s]^{-1}$	Binding of RAS-GTP to SOS(rem)
u_2b	$1\ [s^{-1}]$	Dissociation of RAS-GTP from SOS(rem)
k_2a	$1 \times 10^{-6}/frac\ [s^{-1}]$	RAS activation via SOS-mediated nucleotide exchange; SOS is allosterically upregulated by REM-bound RAS-GDP
k_2b	$k\_2a \times PF\ [s^{-1}]$	RAS activation via SOS-mediated nucleotide exchange; SOS is allosterically upregulated by REM-bound RAS-GTP
b_3	$3 \times 10^{-6}/frac\ [molec \times s]^{-1}$	Binding of RAS-GTP to RasGAP
u_3	$1 \times 10^{-2}\ [s^{-1}]$	Dissociation of RAS-GDP from RasGAP
k_3	$100\ [s^{-1}]$	RAS inactivation via RasGAP induced GTP hydrolysis
b_4	$1 \times 10^{-5}/frac\ [s^{-1}]$	Homodimerization of SOS-free RAS-GTP monomers
b_1	$10^{-1}\ [s^{-1}]$	Dissociation of SOS-free RAS-GTP dimers

**Signal Transduction – RAF/MEK/ERK Cassette**		
**Parameter**	**Value**	**Description**
b_GR	$9 \times 10^{-5}\ [molec\ \times\ s]^{-1}$	Binding of A/B/CRAF to RAS-GTP

u_GR	$100\ [s^{-1}]$	Dissociation of A/B/CRAF from RAS-GDP
u_GR_GTP	$1 \times 10^{-1}\ [s^{-1}]$	Dissociation of A/B/CRAF from RAS-GTP
u_GRR_dim	$10\ [s^{-1}]$	Dissociation of RAF dimers from RAS-GDP
$u\_GRR\_dim\_GTP$	$1\ [s^{-1}]$	Dissociation of RAF dimers from RAS-GTP
b_GRR_dim	$100\ [s^{-1}]$	Homo- and heterodimerization of RAF isoforms on RAS
u_RR_dim	$1\ [s^{-1}]$	Dissociation of RAF homo- and heterodimers not phosphorylated at DIF domain in the cytoplasm
$u\_RR\_dim\_fast$	$u\_RR\_dim \times f\_nfDIM\ [s^{-1}]$	Dissociation of RAF homo- and heterodimers phosphorylated at DIF domain in the cytoplasm
$u\_GRR\_dim\_fast$	$u\_RR\_dim\_fast \times f\_GRR\ [s^{-1}]$	Dissociation of RAF homo- and heterodimers phosphorylated at DIF domain on the RAS platform
$p\_NtA\_dim\_AC$	$1 \times 10^{-1}\ [s^{-1}]$	Phosphorylation of CRAF-S338 and ARAF-S299 within the dimer
$q\_NtA\_cyt\_AC$	$1 \times 10^{-1}\ [s^{-1}]$	Dephosphorylation of CRAF-S338 and ARAF-S299 in the cytoplasm
p_RM	$1 \times 10^{-5}\ [molec\ \times\ s]^{-1}]$	Phosphorylation of MEK on the activation loop by ARAF/BRAF/CRAF
p_M1E	$5 \times 10^{-7}\ [molec\ \times\ s]^{-1}$	Phosphorylation of ERK on the activation loop by MEK1
p_M2E	$p\_M1E \times f\_M2E\ [molec\ \times\ s]^{-1}$	Phosphorylation of ERK on the activation loop by MEK2
q_AL_MEK	$3 \times 10^{-3}\ [s^{-1}]$	Dephosphorylation of MEK1/2 on the activation loop
q_AL_ERK	$1 \times 10^{-2}\ [s^{-1}]$	Dephosphorylation of ERK on the activation loop
q_MP	$100\ [s^{-1}]$	Dephosphorylation of MEK1/2 activation loop after negative feedback phosphorylation

**Signal Transduction – Negative feedback regulation**		
**Parameter**	**Value**	**Description**
p_nfSOS	$3 \times 10^{-8}\ [molec\ \times s]^{-1}$	Feedback phosphorylation of SOS by ERK
q_nfSOS	$1 \times 10^{-3}\ [s^{-1}]$	Dephosphorylation of SOS on the feedback sites
p_nfRAF	$3 \times 10^{-8}\ [molec\ \times s]^{-1}$	Feedback phosphorylation of ARAF/BRAF/CRAF on DIF by ERK
q_nfRAF	$1 \times 10^{-4}\ [s^{-1}]$	Dephosphorylation of ARAF/BRAF/CRAF on the feedback sites

p_nfMEK	$3 \times 10^{-9}\ [molec\ \times s]^{-1}$	Feedback phosphorylation of MEK1 by ERK
q_nfMEK	$3 \times 10^{-4}\ [s^{-1}]$	Dephosphorylation of MEK1 on its feedback site

**Coefficients and Auxiliary Parameters**		
**Parameter**	**Value**	**Description**
EGF_{MM}	300	MM dose coefficient
EGF	Set	Ligand concentration [pg/ml]
EGFs	EGF	Auxiliary parameter necessary for membrane scaling
$Frac$	$EGFs/(EGFs + EGF_{MM})$	The fraction of the membrane undergoing activation
f_M2E	5	The ratio MEK1 and MEK2 catalytic activity
f_M1	$2/3$	The ratio of MEK1 to MEK2
PF	10	Allosteric upregulation of (1) RAS binding to SOS and (2) of RAS GEF-activity by RAS bound to SOS via the Rem domain
f_nfDIM	30	Scaling of dimer dissociation due to phosphorylation of DIF
f_GRR	1×10^{-1}	Scaling of dimer dissociation for dimers bound to RAS platforms

Auxiliary analysis of the model

The impact of the RAS level on system oscillations

RAS and SOS are coupled by a positive feedback loop that introduces bistable switching to the pathway. Not surprisingly, a change in the total RAS level qualitatively influences the activation of RAF isoforms and, thus, downstream components of the MAPK pathway. A tenfold reduction of the total RAS level removes bistability and precludes noticeable RAFs activation (Supplementary Fig. S1). A tenfold increase in the RAS level above the nominal value alters the dynamics, leading to period-doubling and elongation (clearly visible in Supplementary Figs. S1b and S1c). A greater 100-fold increase (with respect to the nominal value) terminates oscillatory behavior because the negative feedback from ERK is insufficient to break the SOS-RAS positive feedback loop and terminate RAS activation (visible in Supplementary Fig. S1a and S1c). For a very high RAS level (beyond physiological limits), RAF dimerization and activation are also reduced because a very high level of RAS dimers reduces the probability that two RAF monomers bind the same RAS dimer, which is needed for RAF dimerization (Supplementary Fig. S2).

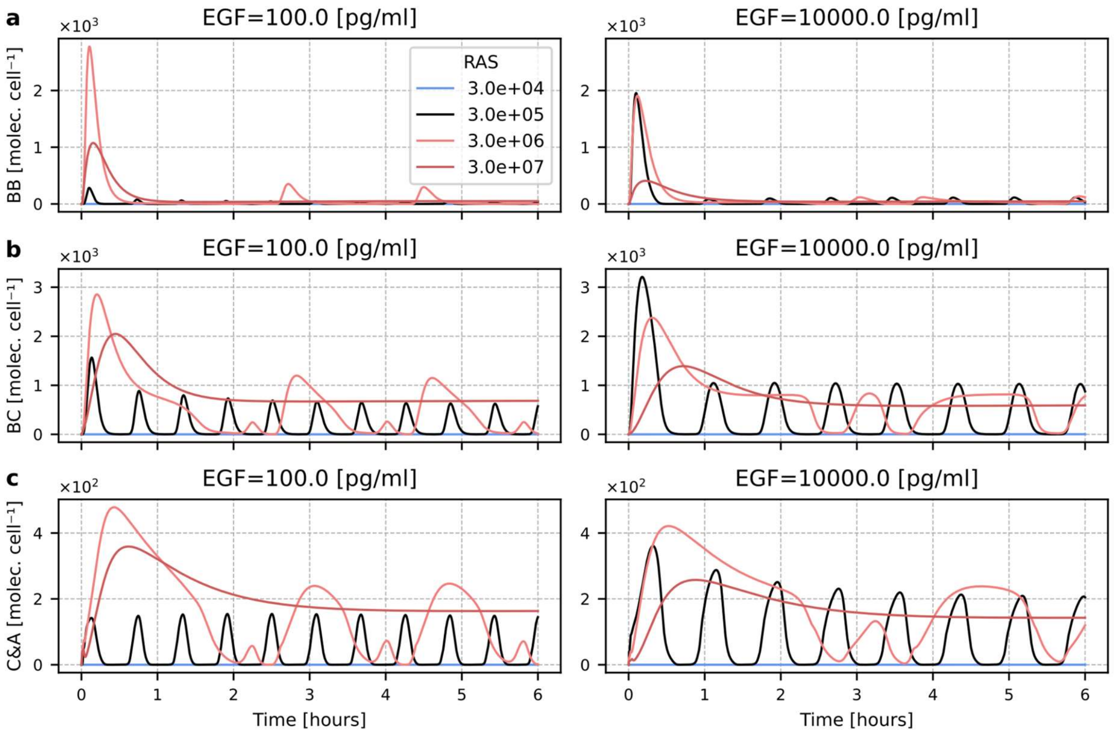

Figure S1. The time profiles of RAF isoform dimers as a function of RAS level.

- **(a)** BB dimers
- **(b)** BC dimers
- **(c)** C&A dimers

Time profiles for two EGF concentrations, 100 and 10000 pg/ml, and four RAS levels. C&A dimers refer to all RAF dimers that do not contain BRAF. The black line corresponds to the nominal RAS level of 3×10^5 molecules per cell.

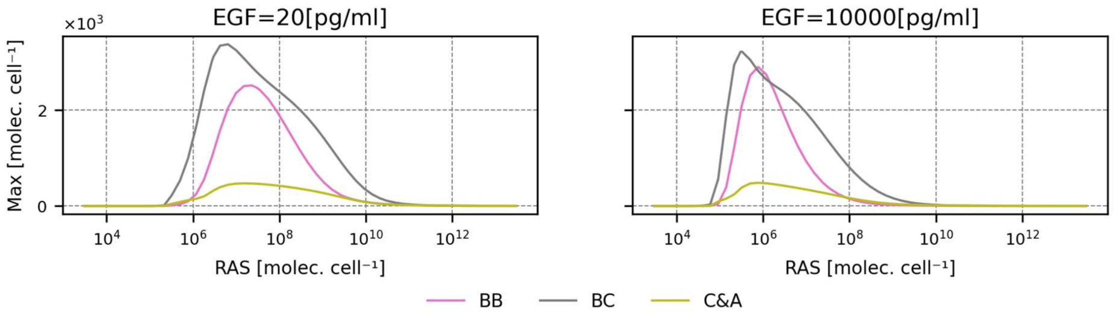

Figure S2. The abundance of RAF isoforms dimers as a function of the RAS level.
Peak values of BB, BC, and C&A dimers as a function of total RAS for low and high EGF concentrations.

Pathway regulation by the MEK isoforms

The ratio of MEK1 to MEK2 determines the strength of the negative feedback from ERK to this tier of the pathway, since MEK1 is the only MEK isoform that possesses an ERK phosphorylation site (Thr292), which phosphorylation downregulates its activity. To the extent that specific functions can be ascribed to individual negative feedback loops, the function of the ERK to MEK feedback is to control the shape of the ERK activity pulse. The ablation of this feedback by setting the MEK1/MEK2 (f_{M1}) ratio equal to zero (Supplementary Fig. S3a) or reducing it by lowering 10-fold the feedback strength (Supplementary Fig. S4a) leads to an almost square-like activity profile, representing the maximal possible level of ERK activity. Increasing feedback strength results in spike-like pulses of ERK activity for low EGF concentrations and broader pulses with reduced ERK activity for high EGF concentrations (Supplementary Fig. S4a). This latter effect can be understood as follows: reduction of MEK activity immediately reduces ERK activity. This reduces ERK's influence on SOS (as well as on RAFs), thereby extending the time to switch off SOS; consequently, we observe longer ERK activity pulses. These extended ERK-PP pulses (observed for increased strength of ERK to MEK feedback) are associated with broader pulses of CRAF in ROKα competent state (Supplementary Fig. S4b) and MST2 incompetent state (Supplementary Fig. S4c).

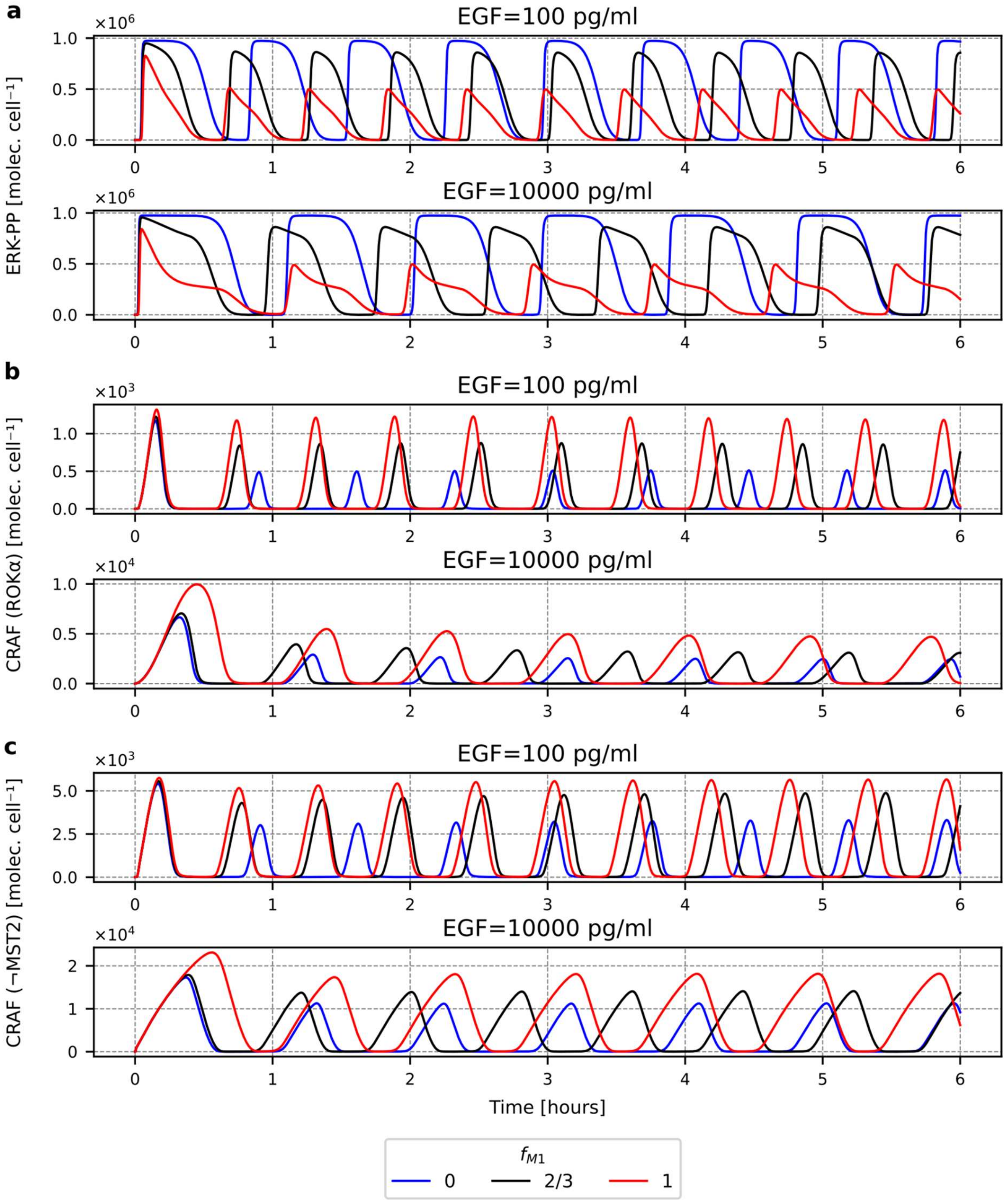

Figure S3. The impact of the MEK1/MEK2 ratio on the time profiles of the pathway components:

- **(a)** ERK-PP
- **(b)** CRAF in ROKα-compatible state
- **(c)** CRAF in MST2-incompatible state.

The black lines correspond to the nominal MEK1/MEK2 ratio f_{M1} of ⅔, blue and red lines correspond, respectively, to MEK1 and MEK2 deficiencies compensated by an increase in the abundance of the second isoform. The corresponding plots for different strengths of ERK to MEK feedback are provided in Supplementary Fig. S4.

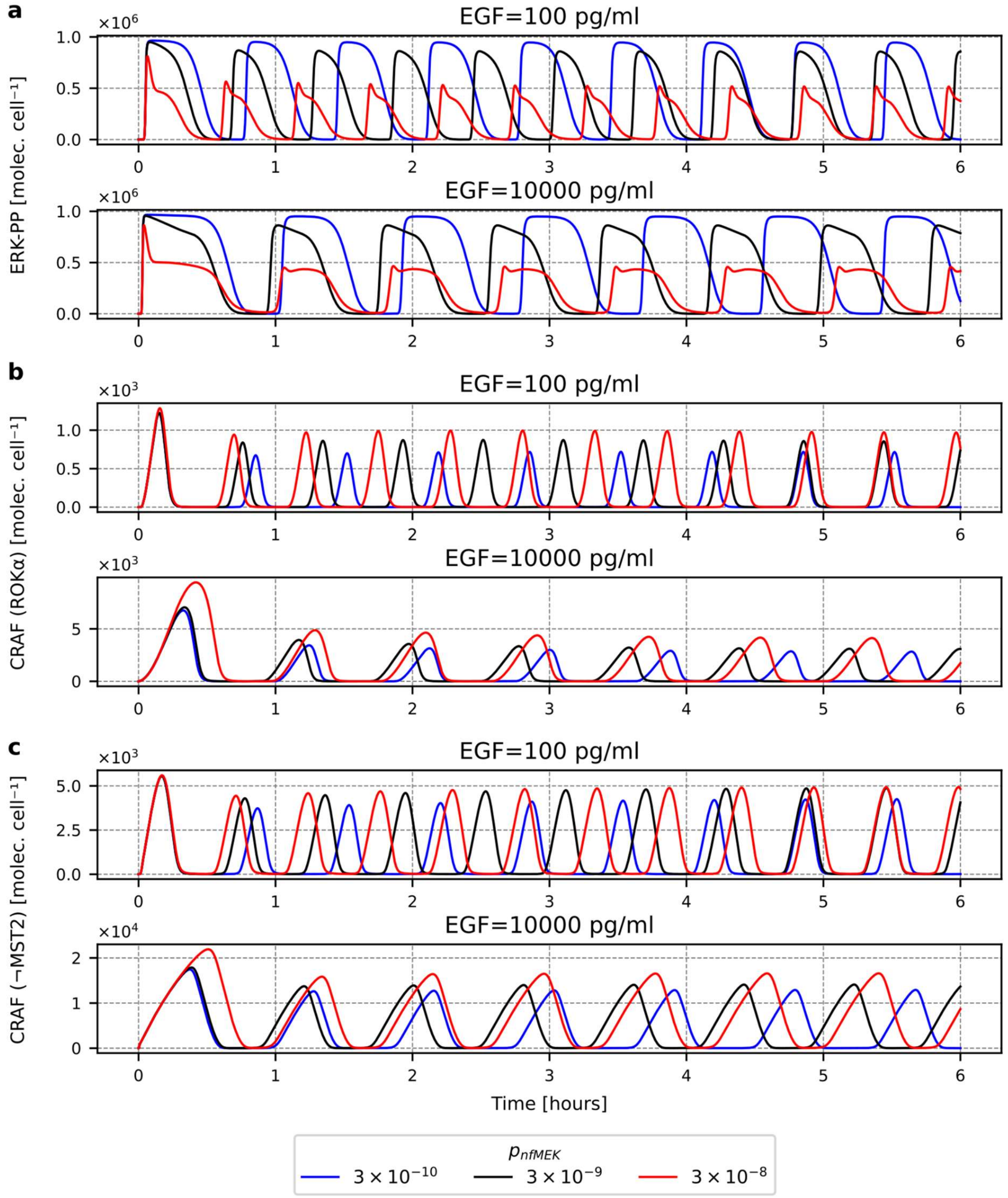

Figure S4. The impact of the ERK to MEK1 feedback strength on the time profiles of the pathway components:

- **(a)** ERK-PP
- **(b)** CRAF in ROKα-compatible state
- **(c)** CRAF in MST2-incompatible state.

ERK-PP, CRAF in ROKα-compatible state, and CRAF in MST2-incompatible state. The black lines correspond to the nominal p_{nfMEK} value, blue and red lines correspond to a tenfold decrease and increase, respectively.

Supplementary References

1. Human Protein Atlas. Preprint at proteinatlas.org.

2. Re3data.Org. The Global Proteome Machine. 484.572 models; 873.636.449 proteins; 9.187.164.998 peptides; 128.620.309.972 residues Preprint at https://doi.org/10.17616/R30C90 (2014).

3. PaxDb: Protein Abundance Database. https://pax-db.org/.

Supplementary Figures S5-S12

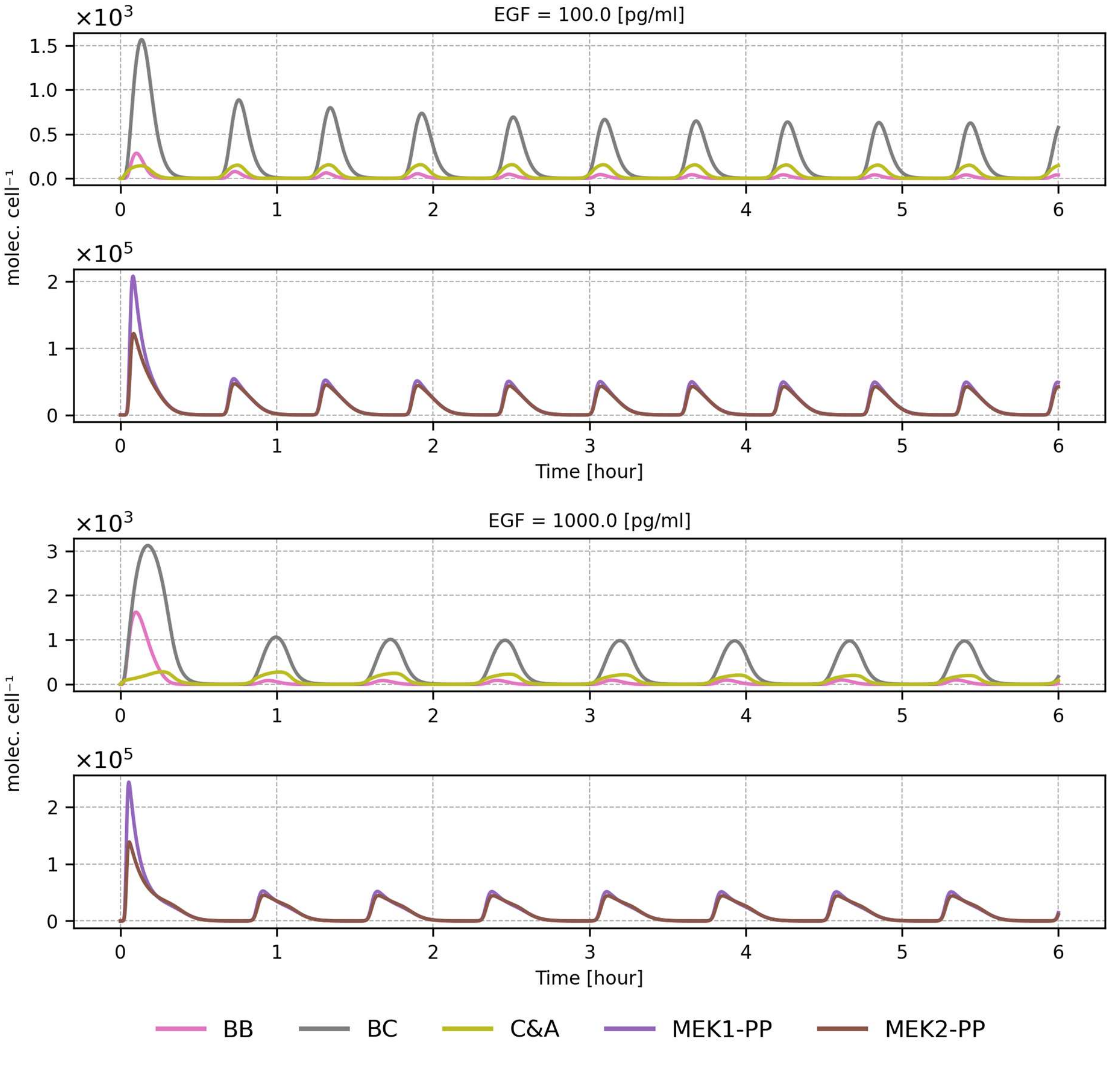

Figure S5. Time profiles of RAF and MEK isoforms during relaxation oscillations. The plot displays abundances of BB, BC, and C&A RAF dimers, as well as MEK1-PP and MEK2-PP molecules for EGF concentrations of 100 pg/ml and 1000 pg/ml. C&A dimers refer to all RAF dimers that do not contain BRAF. The corresponding plots for EGF concentrations of 20 pg/ml and 10000 pg/ml are provided in Fig. 4.

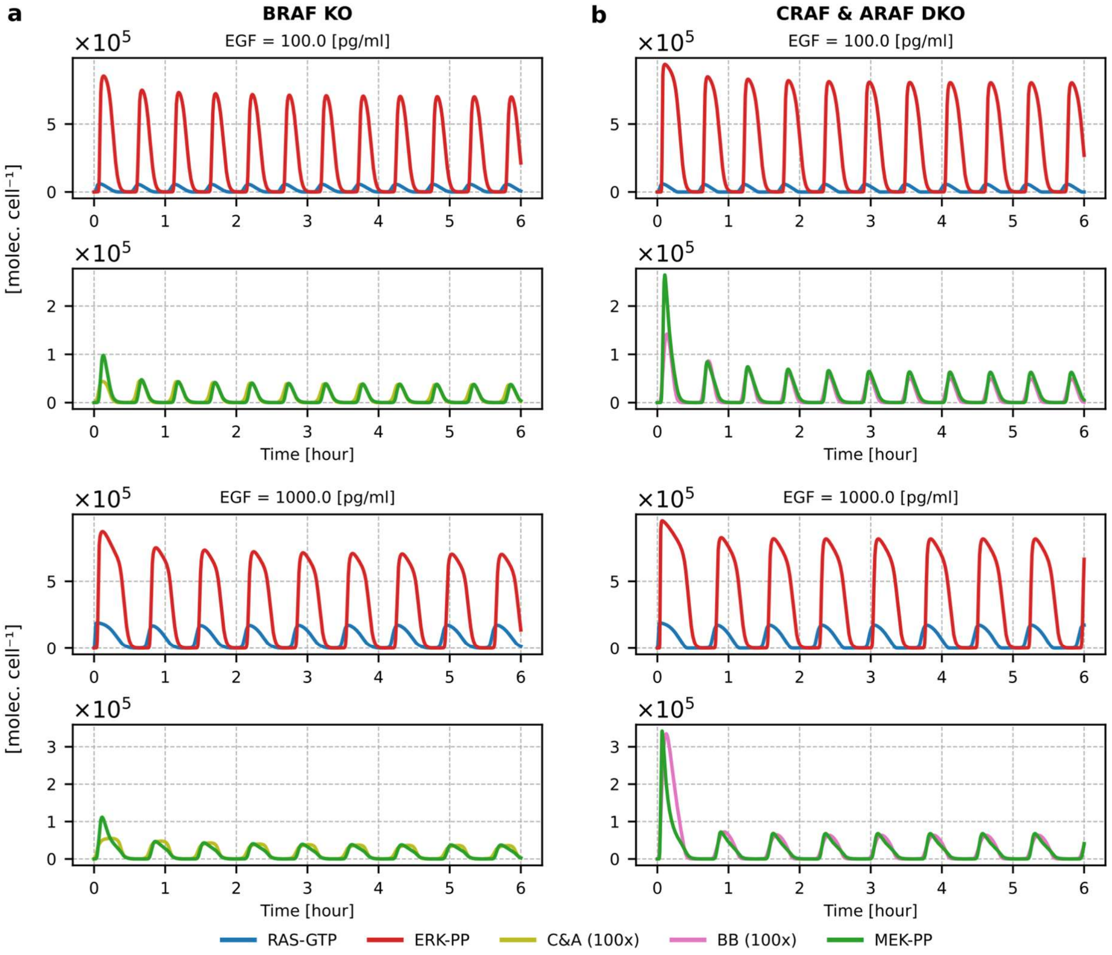

Figure S6. Relaxation oscillations in BRAF KO and ARAF & CRAF DKO cells.

(a) Profiles of RAS-GTP, C&A dimers, MEK-PP, and ERK-PP in BRAF KO cells.

(b) Profiles of RAS-GTP, BB dimers, MEK-PP, and ERK-PP in ARAF & CRAF DKO cells.

The corresponding plots for EGF concentrations of 20 pg/ml and 10000 pg/ml are provided in Fig. 5.

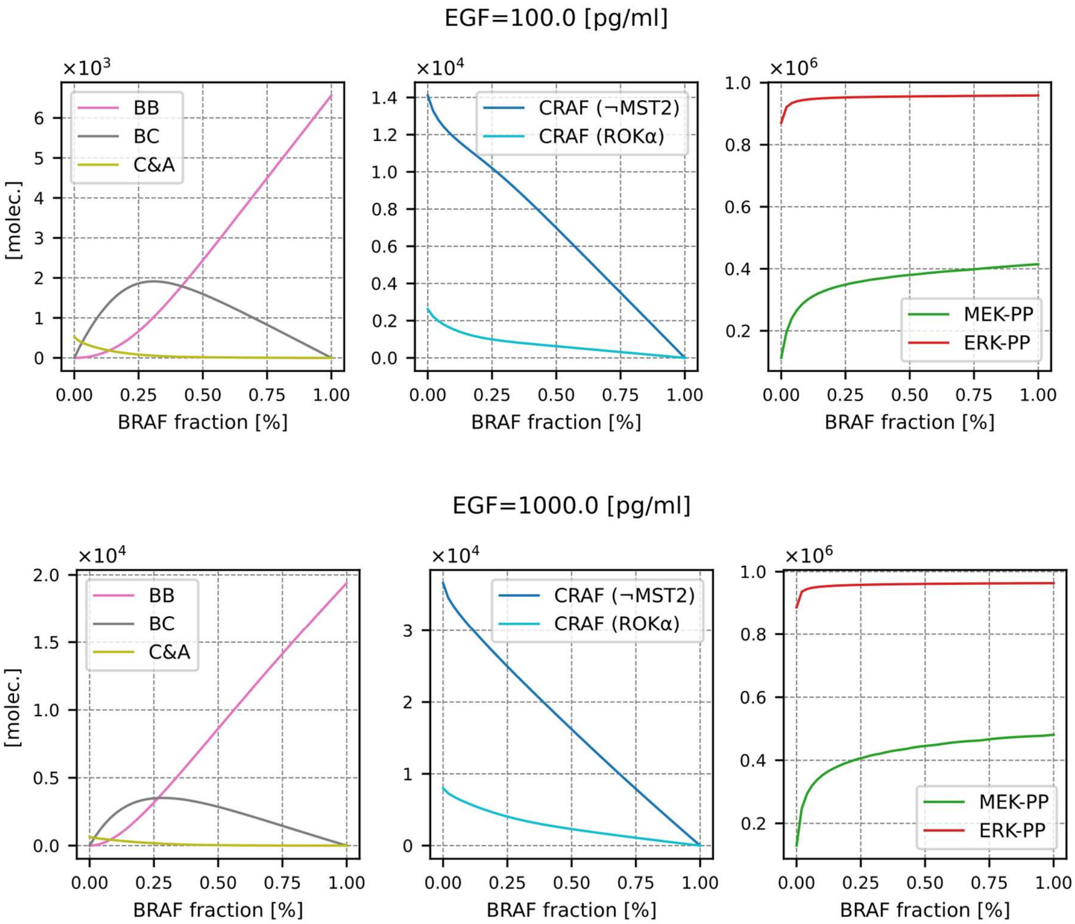

Figure S7. Peak abundances of the main system components as a function of the proportion of BRAF among all RAF isoforms. The total abundance of RAF isoforms is assumed to be constant, and the abundances of CRAF and ARAF are equal. The analysis is performed for EGF concentrations of 100 pg/ml and 1000 pg/ml. The corresponding plots for EGF concentrations of 20 pg/ml and 10000 pg/ml are provided in Fig. 6.

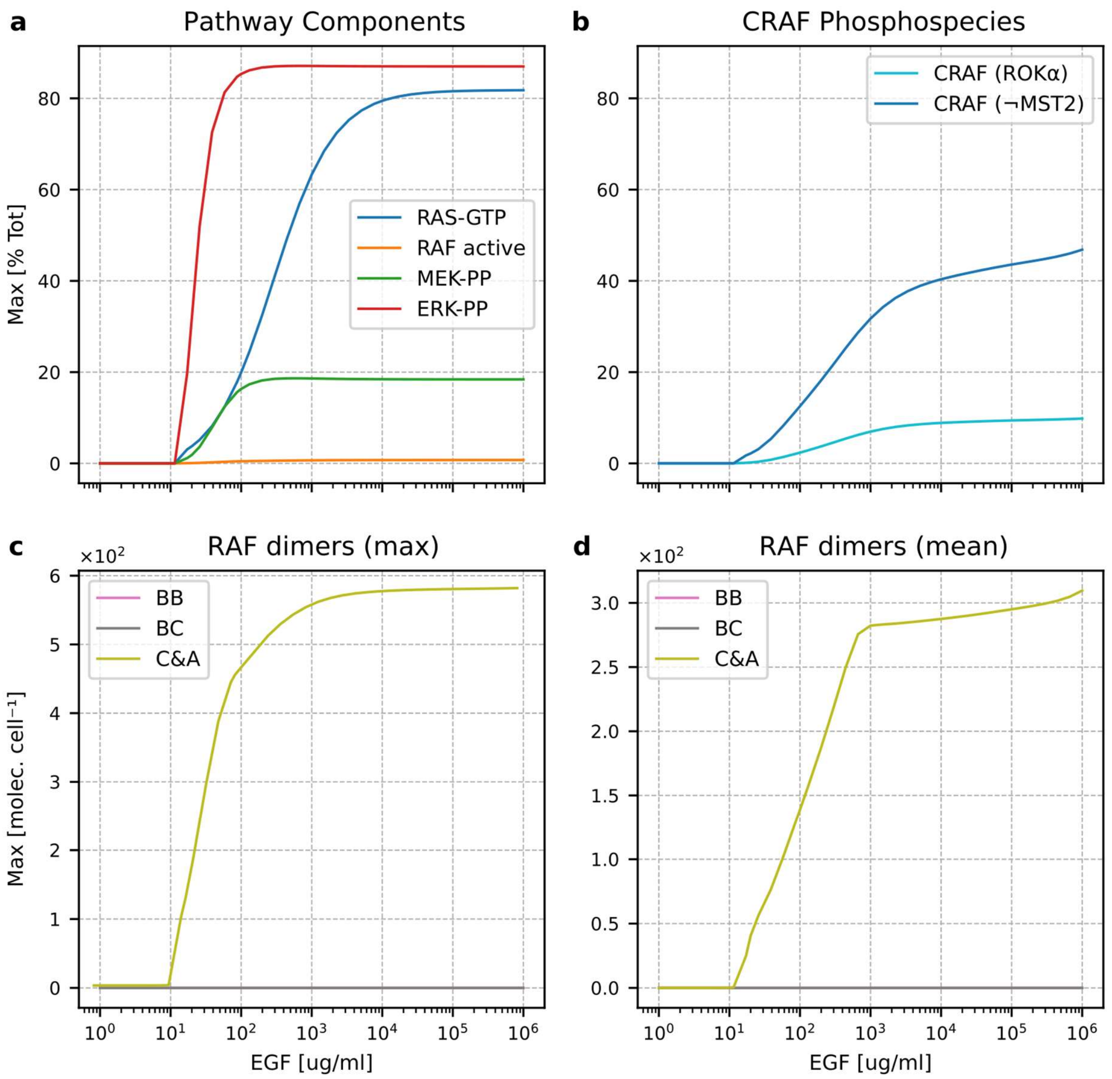

Figure S8. Magnitude of activation of MAPK cascade components in BRAF KO cells as a function of EGF concentration.

- **(a)** The peak values of RAS-GTP, active RAF, MEK-PP, and ERK-PP, as a fraction of total protein level.
- **(b)** The peak values of CRAF (as a fraction of total protein level) in the state in which it can form complexes with ROKα and in the state in which it may not form complexes with MST2.
- **(c)** The peak values of BB, BC, C&A dimers.
- **(d)** The mean values over the first 10 hours of EGF stimulation of BB, BC, and C&A dimers.

The corresponding plots for WT cells are provided in Fig. 7.

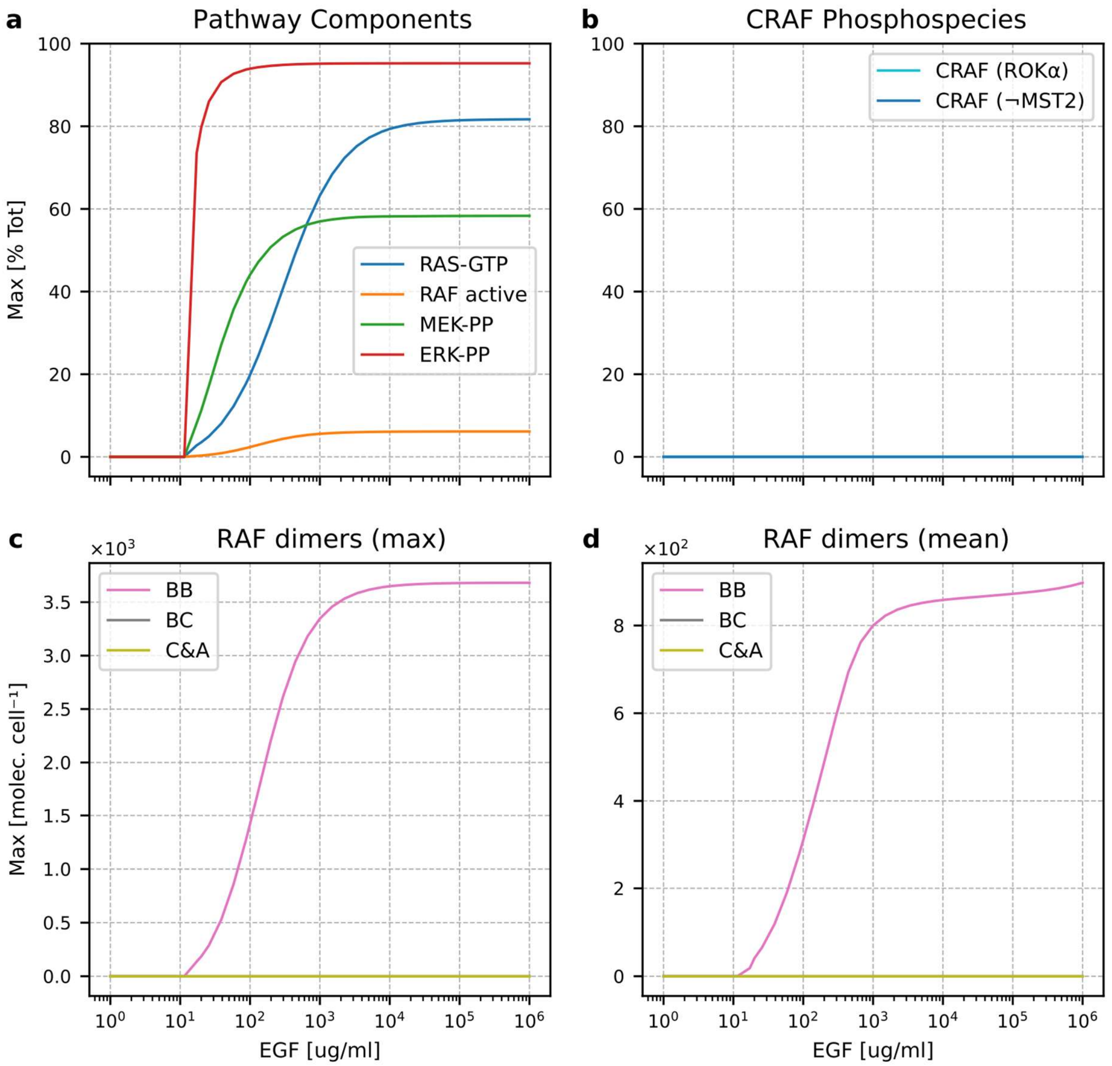

Figure S9. Magnitude of activation of MAPK cascade components in CRAF&ARAF DKO cells as a function of EGF concentration.

- **(a)** The peak values of RAS-GTP, active RAF, MEK-PP, and ERK-PP as a fraction of total protein level.
- **(b)** The peak values of CRAF (as a fraction of total protein level) in the state in which it can form complexes with ROKα and in the state in which it may not form complexes with MST2.
- **(c)** The peak values of BB, BC, C&A dimers.
- **(d)** The mean values over the first 10 hours of EGF stimulation of BB, BC, and C&A dimers.

The corresponding plots for WT cells are provided in Fig. 7.

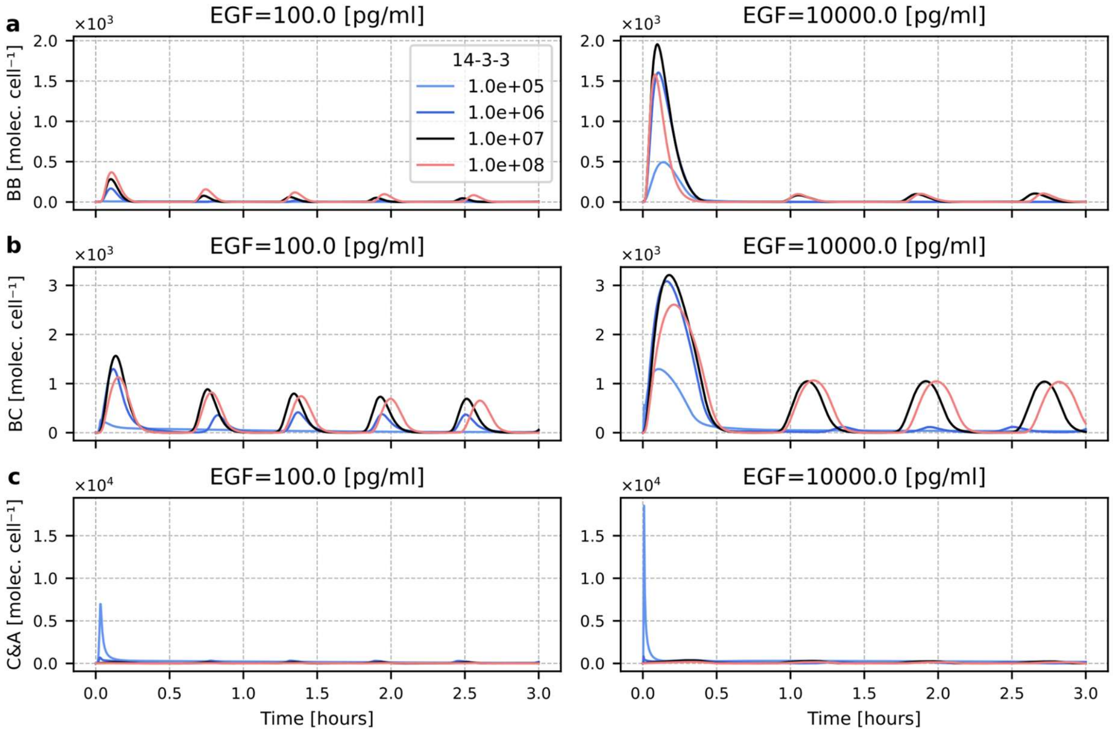

Figure S10. Time profiles of RAF isoform dimers as a function of the 14-3-3 level.

(a) BB dimers

(b) BC dimers

(c) C&A dimers

Time profiles for two EGF concentrations, 100 pg/ml and 10000 pg/ml, and four 14-3-3 levels. The black line corresponds to the nominal 14-3-3 level of 10^7 molecules per cell.

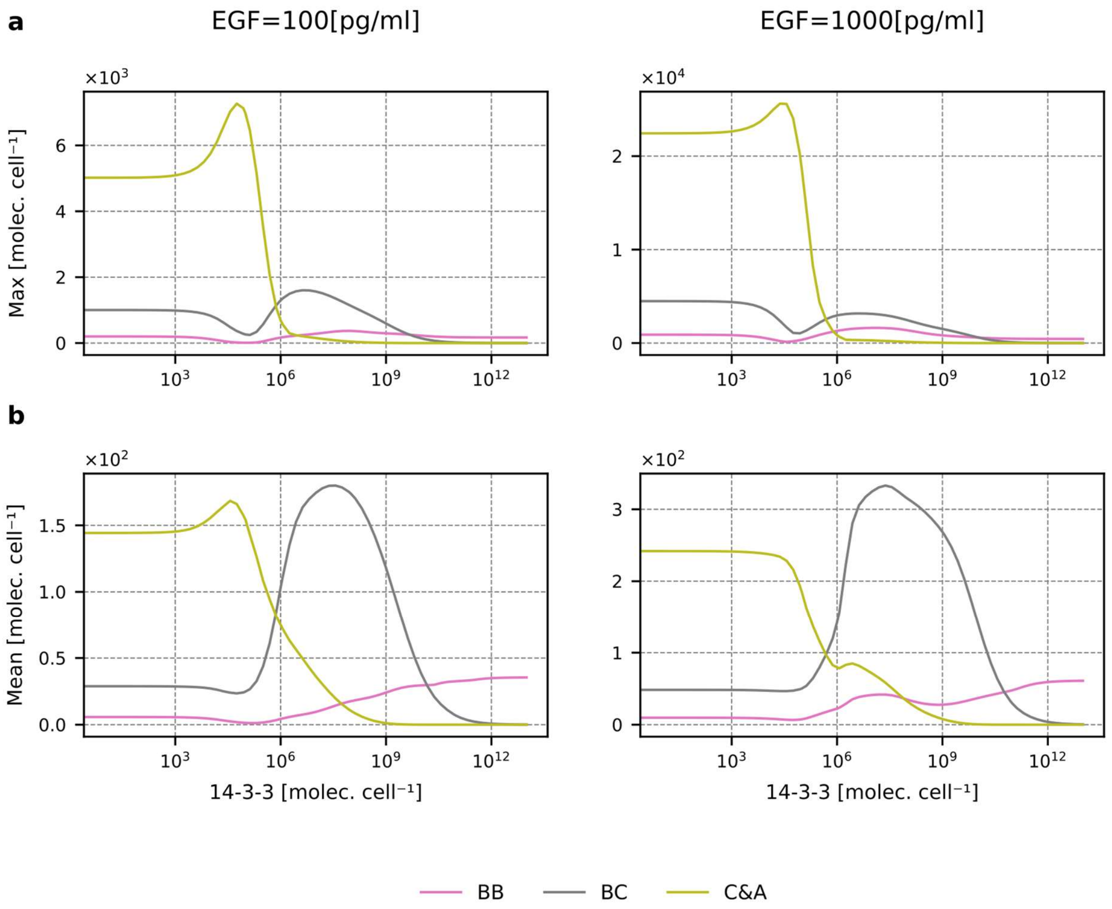

Figure S11. Abundance of RAF isoforms dimers as a function of 14-3-3 level.

(a) The peak value of BB, BC, and C&A dimers as a function of 14-3-3 for EGF concentrations of 100 pg/ml and 1000 pg/ml.

(b) The average value of BB, BC, and C&A dimers (for the first 10 hours of EGF stimulation) as a function of 14-3-3 for EGF concentrations of 100 and 1000 pg/ml.

The corresponding plots for the low and high EGF concentrations of 20 pg/ml and 10000 pg/ml are provided in Fig. 8.

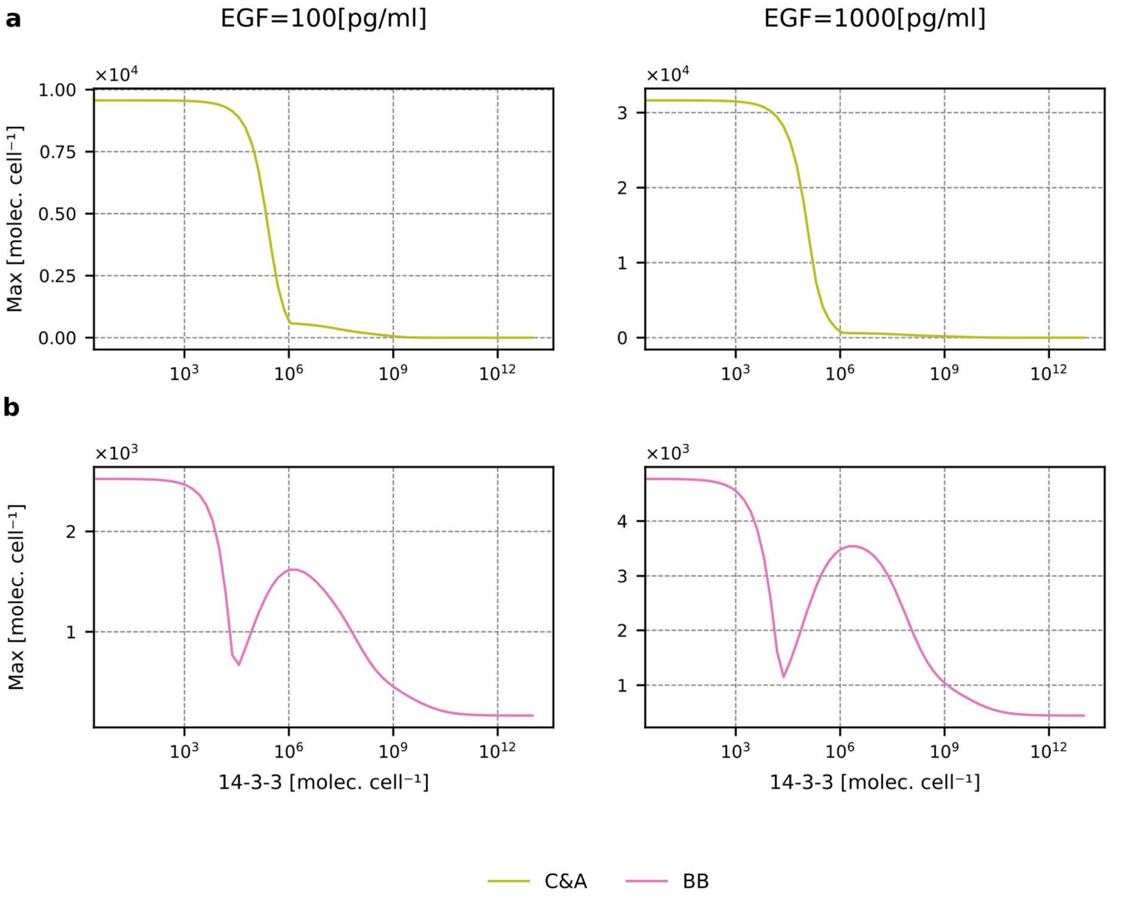

Figure S12. Abundance of RAF isoforms dimers as a function of 14-3-3 level for BRAF KO and CRAF & ARAF DKO cells.

- **(a)** The peak value of C&A dimers in BRAF KO cells as a function of 14-3-3 for EGF concentrations of 100 pg/ml and 1000 pg/ml.
- **(b)** The peak value of BB dimers in CRAF & ARAF DKO cells as a function of 14-3-3 for EGF concentrations of 100 pg/ml and 1000 pg/ml.

The corresponding plots for the low and high EGF concentrations of 20 pg/ml and 10000 pg/ml are provided in Fig. 8.
